# Centromere instability links genome damage to immune activation in systemic sclerosis

**DOI:** 10.1101/2025.08.22.671795

**Published:** 2025-10-30

**Authors:** Azait Imtiaz, Mohammad Waseem, Hudson O’Neill, Bo-Ruei Chen, Wioletta Czaja, Rafael Contreras-Galindo

**Affiliations:** 1 Department of Genetics, University of Alabama at Birmingham, Birmingham, Alabama, 35233, USA; 2 Department of Pediatrics, Washington University in St. Louis, MO, 63001, USA.

**Keywords:** Systemic Sclerosis, Alpha-satellite DNA, DNA double-strand breaks, Bleomycin, Micronuclei

## Abstract

Systemic sclerosis (SSc) is a fibrotic autoimmune disease in which genomic sources of instability and their immunological consequences remain poorly defined. We show that bleomycin, a widely used SSc fibrosis model, induces DNA double-strand breaks (DSBs) at active centromeres. Comparable centromeric damage was detected in fibroblasts from patients with limited cutaneous SSc, with a substantial fraction of cells affected. Quantification of α-satellite repeat length revealed deletions, paralleling alterations observed in diffuse and limited SSc. These breaks are repaired primarily by ATM-dependent RAD51-mediated homologous recombination, but repair remains incomplete. Incomplete repair of centromeric breaks disrupts kinetochore assembly, leading to missegregation and formation of micronuclei and irregular extranuclear chromatin enriched in centromere proteins. BANF1 redistribution was observed on nuclei and micronuclei, and CENP-A/B–positive cytoplasmic chromatin colocalized with MHC class II (HLA-DRB1), suggesting a route for centromeric antigen exposure. Our findings establish BLM exposure as a tractable experimental model for inducing centromere deletions, producing widespread double-strand breaks across active centromeres with signatures consistent with chromatin structure–dependent cleavage. Together, these findings support a model in which centromere instability is an important contributor to chromosomal instability, immune activation, and fibrosis in SSc, providing a mechanistic link between genome instability and autoimmunity.

## Background

Systemic sclerosis (SSc), or scleroderma, is a chronic autoimmune disease characterized by vascular injury, immune dysregulation, and progressive fibrosis of the skin and internal organs [1–4]. A defining clinical feature of limited cutaneous SSc (lcSSc) is the presence of anti-centromere antibodies (ACAs) directed against centromeric proteins such as CENP-A and CENP-B [5,6]. However, the origin of these autoantigens remains unexplained. While recent studies have illuminated downstream immune and fibrotic pathways [7–11], the upstream genomic lesions that precipitate autoimmunity remain largely unknown. Centromeres, complex repetitive regions that govern chromosome segregation, represent a particularly vulnerable genomic compartment whose instability could contribute to the exposure of normally sequestered chromatin antigens to the immune system [2,12].

Human centromeres are composed of ~171-bp α-satellite monomers arranged into higher-order repeat (HOR) arrays. These AT-rich repeats, of which only a subset harbor CENP-B boxes, are packaged into specialized chromatin marked by CENP-A [13,14]. Array size and organization vary extensively across individuals, with up to 30-fold differences recently revealed by near-complete genome assemblies [15]. While centromeres are essential for accurate chromosome segregation, their repetitive nature, secondary structural features, and chromatin context make them prone to replication stress and DNA damage [16–20]. Such fragility positions the centromere as both a guardian and potential trigger of genome instability. Damage in these regions can disrupt kinetochore assembly, drive chromosomal missegregation, and generate micronuclei that serve as compartments for further DNA fragmentation and aberrant repair [17,21–23]. Beyond their structural role, centromeric sequences can thus become active participants in the cellular stress response and, under chronic injury, potential sources of neoantigens.

Missegregated chromosomes frequently form micronuclei that rupture, releasing DNA fragments into the cytoplasm. Cytosolic DNA can activate innate immune sensors, inducing interferon responses [2,24–26]. In fibroblasts, this process coincides with exposure of nuclear proteins such as CENP-A/B and with engagement of MHC class II molecules, particularly HLA-DRB1 alleles associated with ACA-positive SSc [2, 27–30]. These observations suggest that recurrent centromere injury could provide the physical substrate for autoantigen generation in SSc.

Bleomycin (BLM), a chemotherapeutic antibiotic and widely used experimental fibrosis inducer, generates reactive oxygen species that produce both single- and double-strand DNA breaks (DSBs) [31,32]. While a few older studies suggested preferential BLM activity within repetitive DNA, including α-satellite sequences, these reports were limited to single cloned repeats and sometimes considered controversial due to cell-type and assay differences [33–36]. In vivo, BLM administration recapitulates key features of SSc, including dermal fibrosis, fibroblast activation, and extracellular matrix accumulation [37–39]. These properties make BLM a powerful model to test whether centromeric DNA damage drives chromosome instability (CIN), immune activation, and autoantibody generation in SSc [2,40]. We therefore hypothesized that centromeres are preferential targets of BLM-induced DNA damage and that their imperfect repair initiates a cascade leading to CIN and mislocalization of centromeric chromatin for immune recognition.

Despite increasing awareness of repetitive-DNA fragility, the repair dynamics of centromeric DSBs in a fibrotic or autoimmune context remain poorly understood. DSBs can be resolved by non-homologous end joining (NHEJ) or homologous recombination (HR), yet the latter often introduces deletions due to misaligned templates [41–43].

Here, we used BLM-induced DNA damage in fibroblasts and in vivo skin fibrosis models to investigate centromere fragility in the context of SSc. Through α-satellite qPCR, immunofluorescence mapping, ATM inhibition, and patient fibroblast analyses, we show that BLM induces DSBs at active centromeres. These lesions are repaired primarily by RAD51-mediated HR but remain unstable, resulting in DNA loss, kinetochore disruption, chromosome missegregation, and micronucleus formation. Centromeric chromatin became mislocalized to the cytoplasm, coinciding with BANF1 redistribution and colocalization of CENP-A/B with HLA-DRB1. Together, our findings support a centromere-first pathway linking genome instability to antigen exposure and provide a plausible molecular basis for ACA generation in SSc.

## Results

### Centromere instability in BLM-induced SSc fibrosis models

**Fig. 1.**
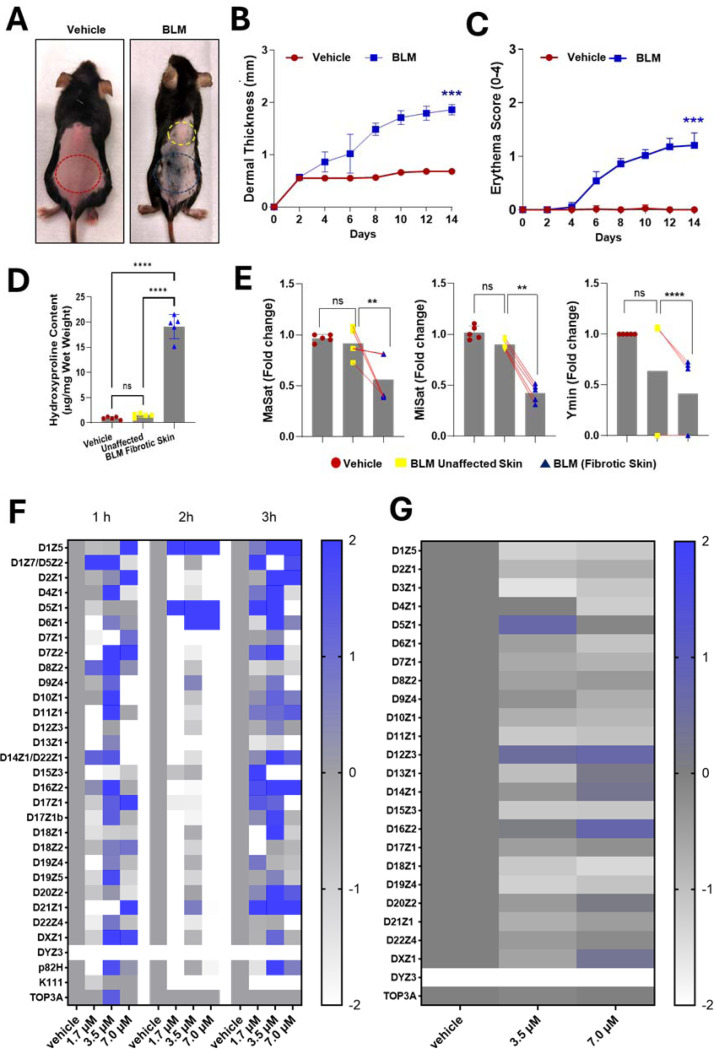
Heatmap and qPCR analysis of α-satellite array abundance in fibroblasts and mouse skin after bleomycin. (A) Representative dorsal skin images of mice collected after 14 days of subcutaneous saline (vehicle) or BLM injection. Red areas denote vehicle-treated control skin, yellow marks adjacent unaffected skin, and blue indicates fibrotic lesions. (B, C) Quantification of dermal thickness (B) and erythema scores (C) in vehicle- and BLM-treated groups (n = 5 per group) showing significant thickening and erythema in BLM-induced lesions. (D) Hydroxyproline quantification in vehicle, unaffected, and fibrotic skin demonstrated increased collagen deposition in fibrotic areas (n = 5 per group). (E) qPCR analysis of mouse centromeric repeat families (MaSat, MiSat, Ymin) normalized to 18S rRNA revealed a marked reduction in α-satellite abundance in fibrotic versus unaffected skin, with red lines connecting paired samples from the same animal; no changes were observed in 18S rRNA levels across groups. (F, G) Heatmaps show log fold changes in human α-satellite monomer abundance across chromosomes in CHON-002 fibroblasts after exposure to BLM for 1–3 h (F) or after 3 h of exposure followed by 24 h recovery (G), normalized to genomic TOP3A as described in Supplementary Fig. 1. Color scale (Z-score of log fold change): blue, gain; white, loss; grey, no change. The Y-chromosomal repeat DYZ3 was absent in CHON-002 cells (female origin). Data represent biological duplicates from three independent experiments; representative results are shown. These analyses demonstrate that both murine and human fibroblasts exhibit centromere-selective reductions in α-satellite DNA following genotoxic stress, reflecting incomplete repair and persistent centromeric instability. Data represent mean ± SD. One-way ANOVA: **p < 0.01; ***p < 0.001; ****p < 0.0001; ns, not significant.

To test whether BLM induces centromere instability in a fibrosis-relevant context, we first used the intradermal BLM mouse skin model, which reproduces dermal thickening, collagen deposition, and fibroblast activation. The mice received BLM injections every other day for 14 days. By day 14, fibrotic skin showed marked dermal thickness, erythema, and hydroxyproline accumulation compared with vehicle control skin (Fig. 1A–D). Quantitative PCR (qPCR) of centromeric repeats revealed significant copy number loss, with deletions of ~50% for MaSat, ~60% for MiSat, and ~70% for Ymin in fibrotic tissue (Fig. 1E). These reductions were consistently observed in all biological replicates (n = 5 mice per group) and correlated with histologic severity (r = 0.82, *p* < 0.01), indicating that centromere depletion scales with fibrosis extent.

In human fibroblasts (CHON-002), BLM exposure (1.7–7.0 μM, 1–3 h) caused a rapid reduction in α-satellite DNA at 2 h, followed by partial recovery at 3 h, consistent with active repair processes (Fig. 1F). At higher doses (7.0 μM), instability persisted. After 24 h of recovery, qPCR revealed sustained copy number alterations (predominantly deletions, with occasional insertions) across multiple α-satellite arrays, including ~50% deletions at D1Z5, D6Z1, D15Z3, and D18Z1 (Fig. 1G). BJ-5ta fibroblasts displayed a comparable pattern but with stronger amplitude of change (−0.8 to −1.5 log fold at several loci; Supplementary Fig. 1A–B).

MTT assays confirmed ~75–80% viability at 3.5–7.0 μM (Supplementary Fig. 2), indicating that centromere copy number changes reflect targeted genomic instability rather than nonspecific cytotoxicity.

Collectively, these results establish that both in vivo and in vitro BLM exposure selectively destabilizes centromeric arrays while maintaining overall cell viability, providing a tractable model to dissect repair dynamics.

### BLM-induced DSBs at active centromeres

**Fig. 2.**
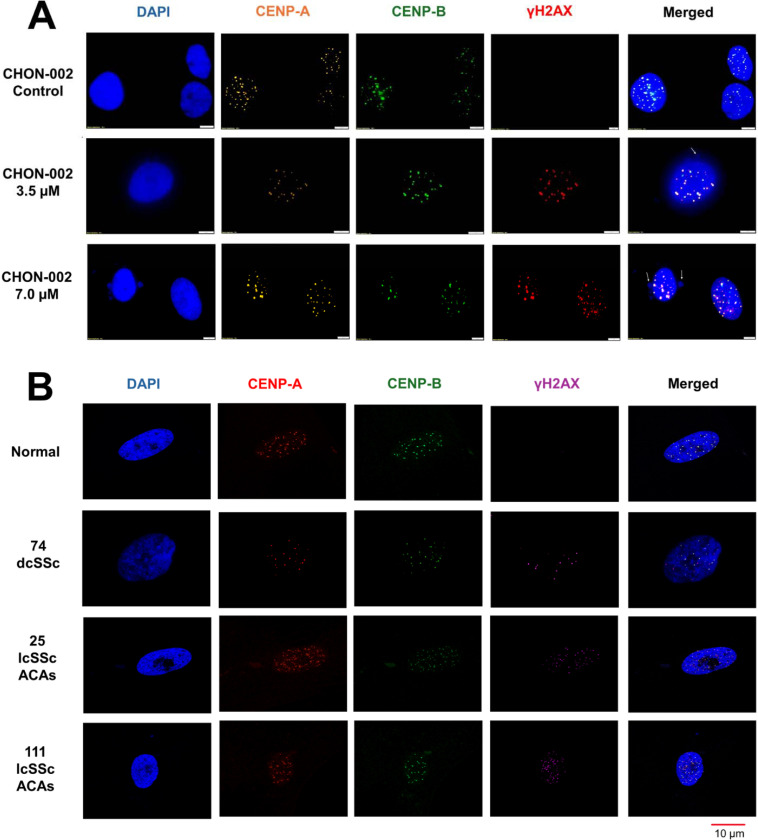
BLM-induced DNA damage at centromere loci in fibroblasts and SSc patient cells. (A) CHON-002 fibroblasts were treated with 3.5 or 7.0 μM BLM for 3 h and stained for CENP-A (orange), CENP-B (green), γH2AX (red), and DAPI (blue). Untreated cells displayed discrete nuclear CENP-A/B foci, whereas BLM exposure produced strong γH2AX staining colocalizing with centromeric regions (CENP-A), indicating accumulation of DSBs at active centromeres. (B) Primary dermal fibroblasts from healthy controls and from patients with diffuse cutaneous (dcSSc) or limited cutaneous (lcSSc) positive for ACAs were stained for CENP-A (green) and γH2AX (purple) with DAPI (blue). Healthy fibroblasts exhibited few γH2AX foci, wherea lcSSc fibroblasts with ACAs showed moderate γH2AX enrichment overlapping with centromeric domains, consistent with persistent centromeric DNA damage in disease cells. Together, these results demonstrate that active centromeres are preferential targets of BLM-induced and disease-associated genome instability.

We next asked whether BLM preferentially induces DSBs at centromeres marked by CENP-A. Compared with untreated controls, CHON-002 fibroblasts treated with 3.5 or 7.0 μM BLM for 3 h showed strong γ-H2AX staining, whereas untreated controls showed no detectable signal (Fig. 2A). The same pattern was observed in BJ-5ta fibroblasts (Supplementary Fig. 3A), and western blot analysis confirmed a robust, dose-dependent increase in γH2AX levels following BLM exposure (Supplementary Fig. 3B).

Colocalization analysis revealed that γH2AX foci frequently overlapped with centromeric markers. While CENP-A and CENP-B signals remained tightly correlated (r > 0.85) under all conditions, γH2AX colocalized more strongly with CENP-A (r = 0.51–0.74) than with CENP-B (r = 0.31–0.50), indicating preferential damage at active centromeres (Supplementary Fig. 3C–D). Double-positive γH2AX/CENP-A foci were not detected in untreated cells but became clearly visible after BLM treatment (*p* < 0.001, n = 50 cells), confirming locus specificity.

To assess disease relevance, we examined fibroblasts from three previously characterized SSc patients analyzed in our prior study [2]. γH2AX activation was observed in all samples, consistent with earlier detection of γH2AX in nuclei and micronuclei of SSc fibroblasts [2]. In fibroblasts from two lcSSc patients, γH2AX colocalized with CENP-A in approximately half of the cells (Pearson’s r = 0.29–0.42), whereas colocalization with CENP-B was lower (r = 0.09–0.19). In contrast, fibroblasts from one dcSSc patient displayed γH2AX foci that rarely overlapped with CENP-A (<1% of cells; Fig. 2B, Supplementary Fig. 3E). These results extend our previous findings by showing that, in addition to γH2AX enrichment at micronuclei, DNA damage also accumulates at centromeric domains within nuclei of lcSSc fibroblasts.

Together, these findings reveal subset-specific patterns of centromeric DNA damage, with ongoing active lesions in lcSSc fibroblasts and more complete structural loss in dcSSc.

### BLM-induced centromeric breaks are preferentially repaired by HR

**Fig. 3.**
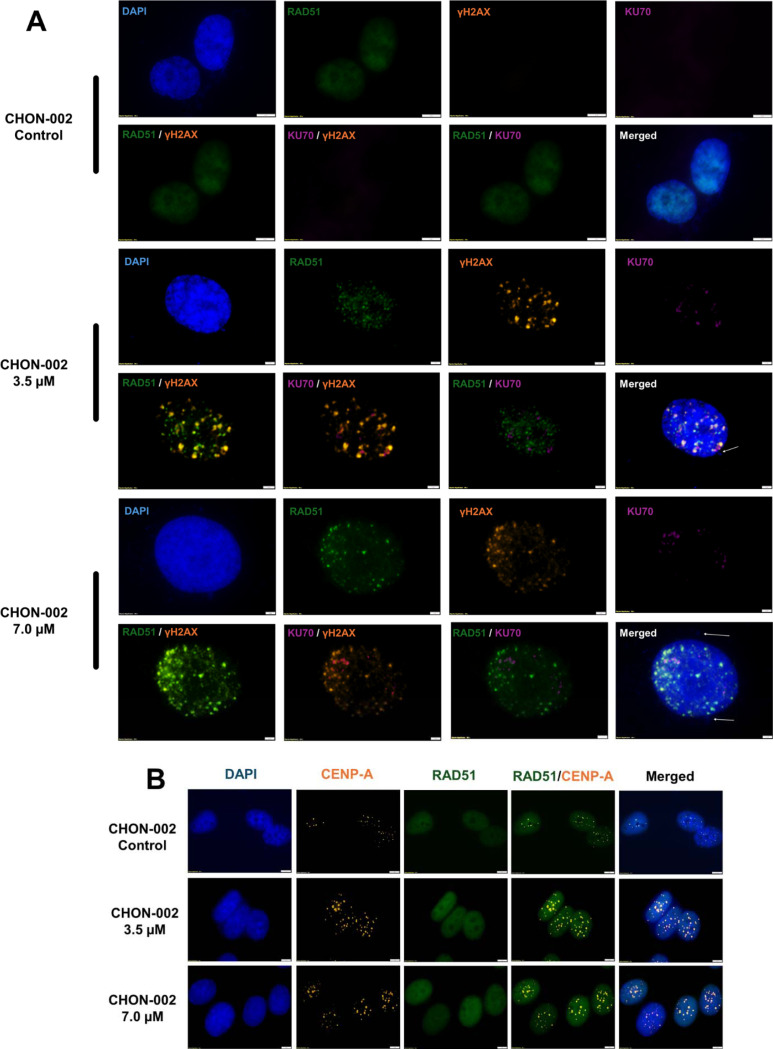
Centromeric DNA repair mechanisms after BLM treatment. CHON-002 fibroblasts were treated with 3.5 or 7.0 μM BLM for 3 h followed by 24 h recovery to assess activation of repair pathways at centromeric lesions. (A) Immunofluorescence (IF) staining for RAD51 (green; HR), γH2AX (orange; DSBs), and KU70 (purple; NHEJ). Untreated controls showed minimal nuclear staining, whereas BLM-treated cells exhibited strong γH2AX foci with prominent RAD51 recruitment at damage sites, indicating engagement of HR at centromeric breaks. White arrows denote micronuclei containing centromeric material. (B) Dual IF for CENP-A (orange) and RAD51 (green) after 3 h of BLM exposure revealed diffuse nuclear RAD51 without discrete foci, consistent with early-stage HR activation prior to focus maturation. Together, these observations indicate that BLM-induced centromeric DSBs activate multiple DNA-repair pathways, including HR and NHEJ, but their incomplete resolution may contribute to persistent centromeric instability.

To define the repair pathways that respond to centromere DSBs, CHON-002 fibroblasts were treated with 3.5 or 7.0 μM BLM for 3 h and stained for γH2AX (DSB marker), RAD51 (HR), and KU70 (NHEJ). Untreated cells showed minimal γH2AX signal, indicating the absence of spontaneous centromeric damage. After BLM treatment, strong nuclear γH2AX foci appeared throughout the nucleus, many overlapping with CENP-A–positive centromeres (Fig. 3A). The γH2AX signal persisted after treatment, confirming the formation of centromeric DSBs that required active repair.

To determine how these lesions were resolved, we next examined RAD51 and KU70. RAD51 recruitment was readily detectable after BLM exposure and localized to the same nuclear regions marked by γH2AX, whereas KU70 staining remained weak and diffuse. Quantitative colocalization confirmed that HR was the predominant repair pathway. At 3.5 μM BLM, the correlation between RAD51 and γH2AX foci was high (r = 0.875), compared with a much lower correlation between KU70 and γH2AX (r = 0.314). At 7.0 μM, RAD51–γH2AX colocalization increased further (r = 0.919; Supplementary Fig. 4A).

Consistent with these imaging data, RT-qPCR showed a strong dose-dependent induction of RAD51 transcripts (*p* < 0.0001), whereas KU70 expression rose modestly (*p* < 0.001; Supplementary Fig. 4B). Over time, RAD51 exhibited a diffuse nuclear pattern at 3 h that resolved into discrete foci by 24 h (Fig. 3B), consistent with ongoing HR and later resolution of repair intermediates.

Together, these findings demonstrate that BLM-induced DSBs at centromeres are recognized by the DNA damage response and repaired primarily through RAD51-dependent HR, with minimal engagement of the NHEJ pathway.

### BLM activates PARP-dependent single-strand break and replication stress signaling

To evaluate whether BLM also induces single-strand breaks (SSBs) and replication stress, we assessed accumulation of the poly(ADP-ribose) (PAR) polymer, a rapid and transient marker of PARP activation. In untreated CHON-002 fibroblasts, PAR immunofluorescence was minimal or undetectable, indicating low basal PARP activity (Supplementary Fig. 4C). After exposure to 7.0 μM BLM, approximately 20% of cells displayed strong, pan-nuclear PAR staining, consistent with acute and transient PARP activation in response to DNA damage. The remaining population showed weak or absent PAR signal, reflecting the short half-life and rapid turnover of PAR polymers. Quantification confirmed a significant increase in nuclear PAR intensity compared with untreated controls (*p* < 0.0001; n = 50 cells; Supplementary Fig. 4D).

Although PAR accumulation indicated activation of the SSB and replication stress response, the predominant DNA lesion induced by BLM was the double-strand break, as evidenced by the widespread γH2AX foci observed under identical conditions.

### ATM-mediated phosphorylation is required for γ-H2AX at centromeric DSBs

**Fig. 4.**
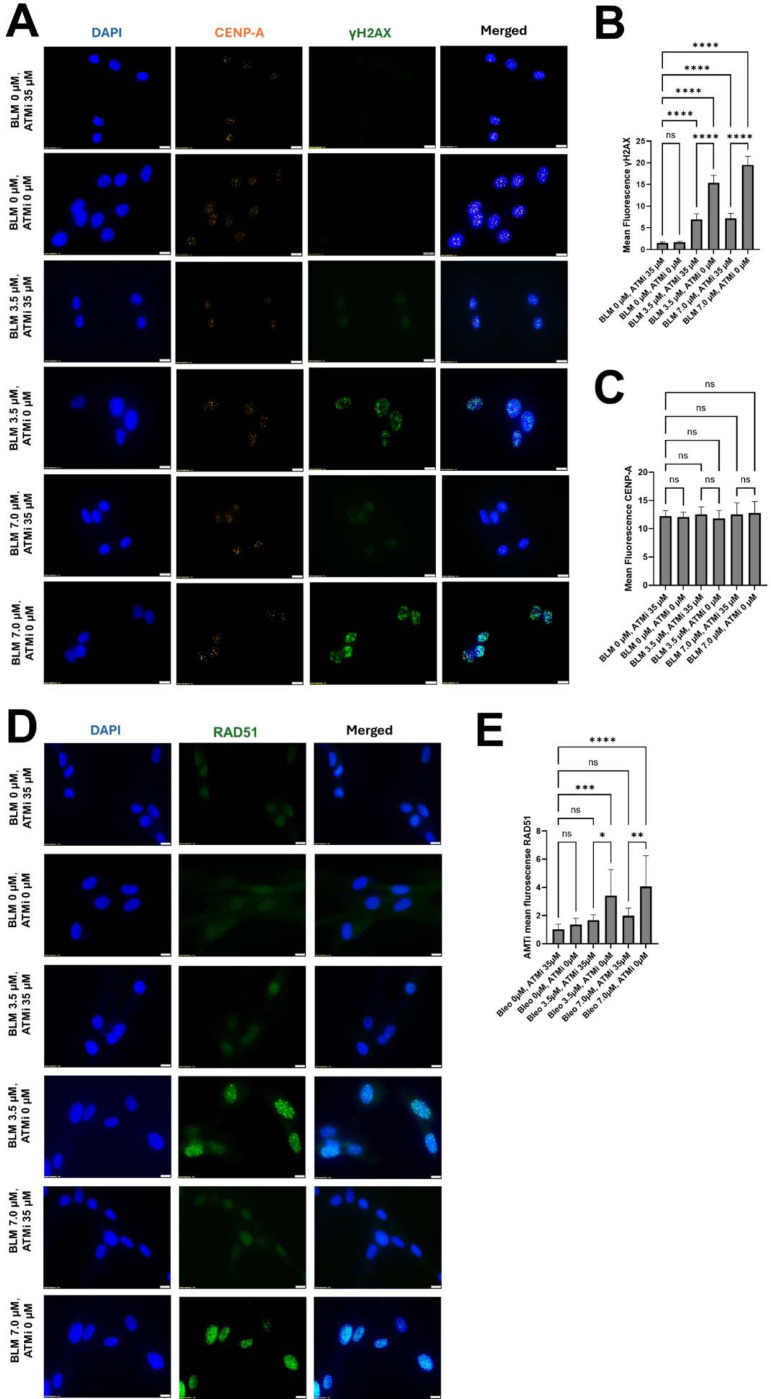
ATM inhibition reduces γH2AX and RAD51 accumulation at centromeres after BLM treatment. CHON-002 fibroblasts were treated with 3.5 or 7.0 μM BLM for 3 h with or without a 1 h pretreatment using the ATM inhibitor KU-55933 (35 μM). (A) Immunofluorescence (IF) for DAPI (blue; nuclei), CENP-A (orange; centromeres), and γH2AX (green; double-strand breaks). BLM exposure induced a dose-dependent increase in γH2AX foci that colocalized with CENP-A, indicating accumulation of DNA damage at active centromeres. ATM inhibition markedly reduced γH2AX accumulation without altering CENP-A localization or intensity. Scale bar, 10 μm. (B) Quantification of mean nuclear γH2AX intensity showing that the dose-dependent induction by BLM was significantly attenuated by ATM inhibition (n = 50 cells per condition). (C) Quantification of mean nuclear CENP-A intensity showing no significant change across treatments. (D) IF for DAPI (blue) and RAD51 (green; HR) demonstrated that ATM inhibition reduced RAD51 recruitment at DNA-damage sites. Scale bar, 10 μm. (E) Quantification of mean nuclear RAD51 intensity confirmed that BLM-induced RAD51 accumulation was significantly suppressed by ATM inhibition (n = 50 cells per condition). Together, these findings demonstrate that ATM kinase activity is required for efficient γH2AX signaling and RAD51 recruitment at centromeric DSBs, linking ATM-dependent signaling to homologous-recombination activation at repetitive DNA. Data represent mean ± SD. One-way ANOVA: *p* < 0.05; *p* < 0.01; *p* < 0.001; *p* < 0.0001; ns, not significant (*p* > 0.05).

ATM phosphorylates histone H2AX on serine-139, generating γH2AX at sites of DSBs. To test whether ATM mediates the centromeric DNA damage signal induced by BLM, CHON-002 fibroblasts were treated with 3.5 or 7.0 μM BLM for 3 h, with or without pretreatment with the ATM inhibitor KU-55933 (35 μM, 1 h). BLM induced a robust, dose-dependent increase in nuclear γH2AX foci, many of which colocalized with the centromeric marker CENP-A (Fig. 4A). Inhibition of ATM substantially reduced both the number and intensity of γH2AX foci at all doses, confirming that phosphorylation of H2AX at centromeric lesions depends on ATM activity. Importantly, KU-55933 treatment did not alter CENP-A localization or abundance (Fig. 4C), indicating that loss of γH2AX reflected impaired signaling rather than centromere disassembly. Across three independent experiments, KU-55933 reduced nuclear γH2AX fluorescence by approximately 70 % relative to BLM-treated controls (*p < 0.001, one-way ANOVA), establishing ATM as the principal kinase initiating the centromeric damage response.

### ATM signaling promotes RAD51 recruitment and HR repair

Because HR predominates at centromeric DSBs, we next examined whether ATM activity influences recruitment of the HR factor RAD51. After BLM exposure, numerous discrete RAD51 foci formed throughout the nucleus and frequently overlapped with γH2AX and CENP-A signals, consistent with assembly of HR repair complexes at damaged centromeres (Fig. 4D). Pretreatment with KU-55933 sharply decreased both the number and fluorescence intensity of RAD51 foci and nearly eliminated their colocalization with γH2AX (Fig. 4D–E). Quantification showed an average 65 % reduction in nuclear RAD51 intensity and a three-fold decrease in cells exhibiting distinct RAD51 foci (*p* < 0.0001, n = 50 cells per condition). These data demonstrate that ATM not only generates the γH2AX signal but also facilitates efficient RAD51 recruitment to centromeric DSBs. In contrast, CENP-A intensity remained unchanged, underscoring that ATM activity modulates repair signaling rather than centromere identity.

Together, these findings define a two-step regulatory role for ATM at centromeric DSBs: first, phosphorylation of H2AX to initiate the DNA damage signal, and second, recruitment of RAD51 to ensure high-fidelity HR repair. Inhibition of ATM leaves centromeric lesions unrepaired, predisposing chromosomes to missegregation and micronucleus formation.

### BLM-induced DNA damage triggers micronuclei formation and centromere chromatin release

**Fig. 5.**
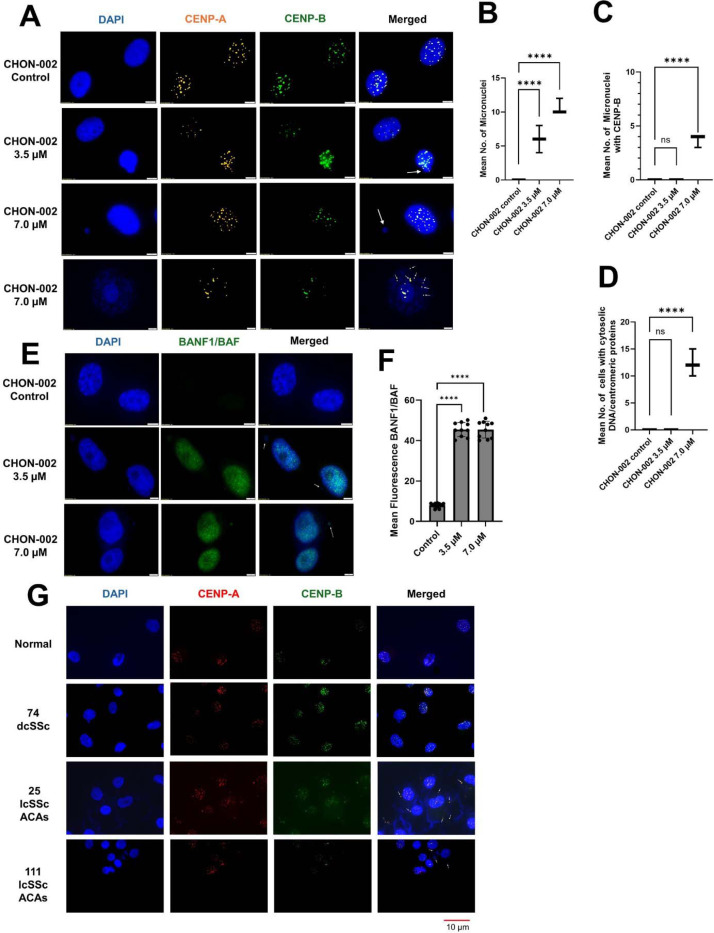
BLM induces micronuclei, cytoplasmic centromeric foci, and nuclear-envelope rupture in fibroblasts and SSc cells. (A) CHON-002 fibroblasts were treated with 3.5 or 7.0 μM BLM for 3 h followed by 24 h recovery and stained for CENP-A (orange), CENP-B (green), and DAPI (blue). Untreated control cells exhibited intact nuclei with discrete centromeric foci, whereas BLM exposure led to frequent formation of micronuclei (white arrows) and cytoplasmic centromeric foci (arrowheads). Scale bar, 5 μm. (B–D) Quantification of (B) micronucleated cells, (C) micronuclei positive for CENP-A/B, and (D) cells containing cytoplasmic centromere-derived protein–DNA structures. Data represent mean ± SD from three independent experiments (n = 100 cells per group). One-way ANOVA: ****p < 0.0001; ns, not significant. (E) Immunofluorescence for BANF1 (green) showed exclusive nuclear localization in control cells but redistribution to both nuclei and micronuclei after BLM treatment (white arrows), consistent with nuclear-envelope disruption. DAPI, blue. Scale bar, 5 μm. (F) Quantification of mean BANF1 fluorescence intensity (n = 10 cells per condition). One-way ANOVA: ****p < 0.0001. (G) Primary fibroblasts from healthy controls and SSc patients stained for CENP-A (red), CENP-B (green), and DAPI (blue). Healthy fibroblasts displayed intact nuclei with discrete centromeric foci, whereas SSc fibroblasts exhibited micronuclei and cytoplasmic centromeric foci (white arrows). Scale bar, 10 μm. Together, these data demonstrate that BLM-induced centromeric instability promotes chromosomal mis-segregation, micronucleus formation, and nuclear-envelope rupture, phenotypes also observed in SSc fibroblasts.

DAPI staining revealed a dose-dependent increase in micronuclei in CHON-002 fibroblasts treated with 3.5 or 7.0 μM BLM, whereas untreated controls showed no detectable micronuclei (Fig. 5A–B). Many of these micronuclei contained centromeric signals identified by CENP-A and CENP-B immunostaining (Fig. 5C, Supplementary Fig. 5E), consistent with previous observations in SSc fibroblasts [2]. In untreated cells, CENP-A and CENP-B localized exclusively to nuclear centromeres, reproducing the organization seen in healthy skin fibroblasts [2]. After BLM exposure, discrete centromeric foci appeared in the cytoplasm, indicating mislocalization of centromeric chromatin. At 7.0 μM, approximately 12% of cells displayed cytoplasmic centromere chromatin (*p* < 0.0001, n = 3; Fig. 5D), a finding also observed in BJ-5ta fibroblasts (Supplementary Fig. 5A–D). Cytoplasmic DNA was detected in ~15% of cells at this dose (*p* < 0.0001, n = 3), consistent with BLM being associated with cytoplasmic mislocalization of centromeric chromatin and DNA.

To test whether nuclear envelope rupture mediates this release, we examined the localization of BANF1, which we previously showed to accumulate at rupture sites in lcSSc fibroblasts with cytoplasmic centromere leakage [2]. Consistent with those observations, BLM-treated fibroblasts displayed strong BANF1 accumulation on disrupted nuclei and micronuclei, consistent with nuclear-envelope disruption at nuclei and micronuclei associated with centromere-containing chromatin mislocalization (Fig. 5E–F).

Patient fibroblasts presented similar phenotypes [2]. Fibroblasts from lcSSc patients frequently displayed cytoplasmic centromeric chromatin, whereas those from dcSSc patients rarely did (Fig. 5G). This pattern correlates with the higher prevalence of ACAs among lcSSc patients. In our prior analyses, ACAs were detected in 100% of patients showing cytoplasmic centromere chromatin. Taken together, these data, along with BANF1 redistribution, implicate nuclear envelope rupture as a mechanism driving centromeric chromatin release in lcSSc fibroblasts.

### Centromere damage drives cytoplasmic mislocalization and MHC class II presentation

Cytoplasmic mislocalization of centromeric chromatin was a defining feature of fibroblasts treated with BLM, consistent with the phenotype previously documented in ACA-positive lcSSc fibroblasts [2]. In our earlier study, we detected cytoplasmic staining of the centromere proteins CENP-A and CENP-B in six of nine lcSSc patient fibroblast cultures, ranging from 4 to 78 percent of cells (n = 500). This phenotype correlated completely with the presence of anti-centromere antibodies and was absent in fibroblasts from healthy controls, dcSSc patients, and ACA-negative lcSSc fibroblasts. We now show that this mislocalization can be mechanistically reproduced by BLM-induced centromere damage in otherwise normal fibroblasts, indicating that genotoxic stress alone is sufficient to trigger cytoplasmic release of centromeric chromatin.

**Fig. 6.**
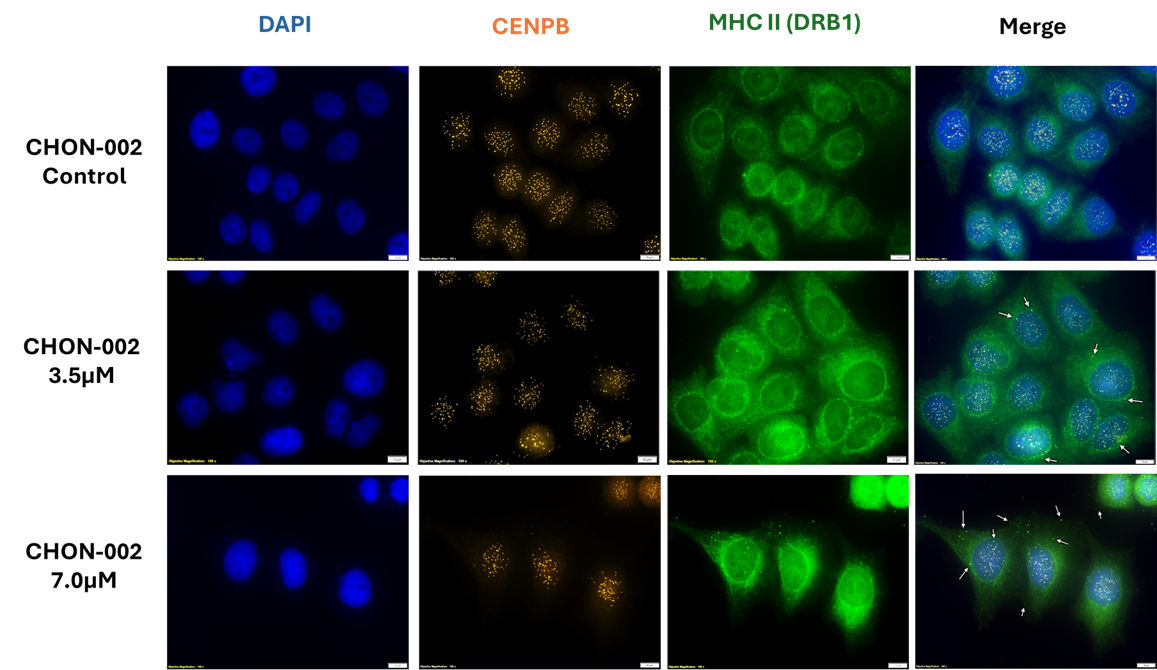
Colocalization of cytoplasmic CENP-B and MHC class II (DRB1) after BLM treatment. CHON-002 fibroblasts were left untreated or treated with 3.5 or 7.0 μM BLM for 3 h followed by 24 h recovery, then stained for CENP-B (orange) and MHC class II (DRB1, green) with DAPI (blue) marking nuclei. Untreated control cells showed no cytoplasmic overlap between CENP-B and MHC class II, whereas BLM-treated fibroblasts displayed distinct cytoplasmic regions where CENP-B and MHC class II colocalized (white arrows). Scale bar, 10 μm. These findings suggest that centromeric chromatin fragments released into the cytoplasm after DNA damage can associate with antigen-presentation machinery, potentially linking centromeric instability to immune activation.

To determine whether this cytoplasmic centromere material interacts with antigen presentation pathways, we performed immunofluorescence staining for CENP-B and MHC class II molecules (HLA-DRB1). BLM-treated fibroblasts exhibited strong cytoplasmic colocalization of CENP-B and HLA-DRB1, whereas untreated controls showed neither cytoplasmic centromere chromatin nor colocalization (Fig. 6). The overlap was confined to cells displaying cytoplasmic CENP-B foci, consistent with a direct relationship between chromatin mislocalization and engagement of the antigen presentation machinery.

This phenomenon closely parallels what we observed in ACA-positive lcSSc fibroblasts, where CENP-B colocalized with HLA-DRB1 and HLA-DRB5 in the cytoplasm and along the plasma membrane. These interactions were absent in fibroblasts from dcSSc patients and healthy individuals [2]. The parallels between disease-derived cells and the BLM model indicate that persistent centromere damage and nuclear envelope rupture provide a physical route for centromere-derived chromatin to encounter MHC class II molecules.

Together, these findings show that BLM-induced centromere instability recapitulates a key immunopathological feature of lcSSc: the cytoplasmic exposure and potential MHC-II presentation of centromeric antigens. This establishes a direct mechanistic connection between genotoxic stress, nuclear rupture, and the initiation of centromere-targeted autoimmunity.

## Discussion

This study identifies centromeric DNA as a critical target of genotoxic stress in SSc. We demonstrate that BLM-induced breaks within centromeric α-satellite arrays are signaled through ATM-dependent γH2AX and repaired by RAD51-mediated HR, but the lesions remain incompletely resolved, resulting in deletions or abnormal insertions in α-satellite DNA. We observed DSB formation across nearly all active CENP-A marked centromeres, indicating a genome-wide centromere response rather than isolated sequence defined hotspots. This pattern suggests a chromatin structure-dependent, non-sequence-specific cleavage mechanism that reflects the unique DNA topology and protein DNA organization of active centromeres. These findings help clarify long-standing uncertainty about whether BLM targets α-satellite DNA in intact cells. A key consequence of this damage is cytoplasmic colocalization of centromere chromatin, where it colocalizes with MHC class II molecules and may provide a direct source of anti-centromere autoantigens. Parallel findings in patient fibroblasts and in the BLM-induced skin fibrosis model establish centromere instability as a disease-relevant driver of immune activation and fibrosis.

### BLM as a model for centromere deletions

Our data support the use of acute BLM exposure as a general and controllable model to study centromere deletions and DNA repair pathway choice. Because BLM triggers DSBs across active centromeres without obvious sequence bias, it allows systematic investigation of how chromatin state, replication timing, and repair factor availability influence deletion spectra and reintegration outcomes at α-satellite arrays. This centromere-first stress model should be useful beyond SSc, including other conditions where centromere instability, micronuclei formation, or antigen exposure are involved, such as cancer and fibrosing disorders. Similar large-scale rearrangements arising from micronuclear chromosome fragmentation, such as chromothripsis [24], may share mechanistic overlap with centromere breakage events.

### Centromere instability as an immunologically active process

Our results reposition centromere instability from a passive structural defect to an active immunologic mechanism. The discovery that damaged centromere chromatin can exit the nucleus and engage with the MHC class II pathway provides a clear molecular link between chromosomal instability and systemic autoimmunity [44,45]. The same sequence features that ensure faithful chromosome segregation, including repetitiveness, transcriptional silencing, and heterochromatin compaction, also make these regions vulnerable to replication stress and error-prone repair. Once disrupted, their repetitive structure may amplify both genomic instability and antigenic exposure.

Centromeric repeats are intrinsically fragile because of their repetitive composition, replication challenges, and specialized chromatin environment. Previous studies showed that centromeres accumulate spontaneous lesions during both proliferation and quiescence and are most often repaired through RAD51-mediated HR [18,20,46]. In our experiments, BLM strongly amplified this baseline fragility, producing deletions and insertions within α-satellite arrays. Alterations were more pronounced in BJ-5ta fibroblasts than in CHON-002 cells, suggesting that chromatin state or repair capacity influences centromeric vulnerability. The BLM-induced skin fibrosis model reproduced these effects *in vivo*, showing consistent reductions in mouse centromeric satellite repeats within fibrotic tissue. These results indicate that centromere instability is not an incidental by-product of DNA damage but a defining molecular feature of fibrosis. Together, these observations support BLM as a discovery platform for the study of centromere deletion formation and maintenance.

γH2AX foci colocalized with CENP-A, and ATM inhibition suppressed this signal, showing that centromeric lesions rely on ATM-dependent signaling. Recruitment of CENP-A to double-strand breaks has been observed previously in mammalian cells [47], supporting the idea that centromeric chromatin participates directly in DNA-damage signaling and repair. Recruitment of RAD51, but not KU70, indicated a preference for HR repair, although this process did not completely restore array integrity [20,46,48]. Incomplete HR may therefore underlie the characteristic α-satellite deletions detected in SSc fibroblasts, which is consistent with reduced centromere signal intensity observed in SSc skin biopsies [2]. Because CENP-A is essential for centromere identity and kinetochore assembly [49], persistent disruption of this domain would be expected to impair chromosome segregation and stability.

### Cytoplasmic fate and antigen presentation

Centromere instability is associated with chromosome missegregation, micronucleus formation, signs of nuclear-envelope disruption (BANF1 redistribution), and cytoplasmic mislocalization of centromeric chromatin. These cytoplasmic structures appeared in about 12 to 15 percent of treated fibroblasts and mirrored phenotypes observed in lcSSc fibroblasts. We observed robust cytoplasmic colocalization of CENP-B with HLA-DRB1, consistent with engagement of the MHC class II pathway. We did not assess peptide loading or antigen specificity; therefore, MHC-II colocalization supports potential engagement of antigen-presentation pathways without demonstrating presentation of centromere-derived peptides.

One mechanistic model is that euchromatic centromere-proximal domains, corresponding to active CENP-A chromatin, are cleaved and mobilized into the cytoplasm where they encounter antigen-presenting machinery, whereas heterochromatic pericentromeric regions may remain tethered or be released as larger, less processed chromatin fragments that fail to reintegrate efficiently. These alternatives, fragmented export and bulk chromatin release, are not mutually exclusive and may vary with damage load and nuclear envelope integrity [2,5,50–53].

The difference between lcSSc and dcSSc fibroblasts observed here, with active centromere fragility in the former and more complete deletions in the latter, may help explain the distinct immunologic outcomes of these subsets. Persistent low-level instability could continuously supply neoantigens in lcSSc, promoting anti-centromere antibody production, whereas more catastrophic centromere loss in dcSSc might drive fibrosis without sustained autoantibody generation. These inferences are based on a small number of primary cultures and may be influenced by inter-line variability; larger cohorts will be required to confirm subset-specific mechanisms. These results raise the possibility that the extent and nature of centromere damage could stratify patients by their dominant pathogenic mechanism [5,27,28].

### Limitations and future directions

Our conclusions rest on convergent lines of evidence, including qPCR and heatmap analyses of α-satellite loss, γH2AX–CENP-A colocalization, ATM- and RAD51-dependent repair, micronucleus and nuclear envelope rupture phenotypes, and CENP-B colocalization with HLA-DRB1 across cell lines, primary fibroblasts, and the BLM skin model. As with any reductionist system, acute BLM exposure does not capture all features of chronic tissue remodeling, but it provides a reproducible framework for probing centromere damage and repair outcomes. Future studies should define precise break locations within active centromeres, determine how repair pathway choice shapes deletion patterns, clarify how centromeric chromatin engages the MHC class II pathway, and evaluate whether cytoplasmic centromeric DNA activates cGAS or downstream innate and cellular immune pathways that contribute to fibroblast activation and antigen presentation [25], these efforts will build upon, rather than alter, the central conclusions presented here.

## Concluding remarks

Our data support a centromere-first model in which repetitive DNA damage initiates fibrosis and autoimmunity. This framework links genome instability, defective repair, and antigen exposure in a single continuum of disease progression. By showing that BLM provokes global double-strand breaks at active centromeres through a structure-dependent mechanism, followed by cytoplasmic export and MHC class II association, we provide a general experimental system to study centromere deletions and their immunologic consequences. Because repetitive DNA is abundant throughout the genome, similar processes may occur in other autoimmune or fibrotic diseases that compromise nuclear integrity. Understanding how the most conserved regions of the human chromosome become unexpected sources of immune activation opens new possibilities for therapeutic strategies that enhance DNA repair fidelity and protect chromatin integrity.

## Materials and methods

### Reagents

Bleomycin sulfate (Cayman Chemical, Cat. No. 13877, Lot #0701763–49) was used in all experiments to ensure consistency across assays. All other reagents were of analytical grade and obtained from commercial suppliers unless otherwise noted. Phosphate-buffered saline (PBS; pH 7.4), paraformaldehyde (PFA), and Triton X-100 were from Thermo Fisher. Bovine serum albumin (BSA; Sigma) was used for blocking at 2% (w/v). ProLong Gold Antifade Mountant with DAPI (Thermo Fisher) was used for all imaging.

### Cell culture and treatments

hTERT-immortalized human fibroblasts (CHON-002, ATCC CRL-2847; BJ-5ta, ATCC CRL-4001) were cultured in Dulbecco’s Modified Eagle Medium (DMEM; Gibco) supplemented with 10% fetal bovine serum (FBS; Gibco) and 1% penicillin–streptomycin (Gibco). The cells were maintained at 37 °C in a humidified 5% CO incubator and routinely tested for mycoplasma contamination.

For acute bleomycin (BLM) exposure, fibroblasts were seeded at 5 × 10 cells per well in 6-well plates and grown to 60–80% confluence. The cells were treated with 0, 3.5, or 7 μM BLM for 3 h. For recovery assays, cells were washed with PBS and incubated in drug-free medium for 24 h. For ATM inhibition, cells were pretreated with KU-55933 (35 μM, Selleck Cat. S1092) for 1 h prior to BLM exposure.

### Mouse BLM-induced skin fibrosis model

C57BL/6 mice (Jackson Laboratory; 20–25 g; 2 males, 3 females per group) were randomized to receive intradermal injections of BLM (1 U/mL, 100 μL) or vehicle (PBS) into shaved dorsal skin every other day for 14 days. On day 14, mice were anesthetized with isoflurane (1–2%) and euthanized by cervical dislocation. Skin samples were collected from (1) PBS-injected control sites, (2) adjacent uninjected skin, and (3) fibrotic BLM-injected lesions. Dermal thickness was measured from H&E sections at 10 random fields per mouse using Fiji (ImageJ v1.54). Hydroxyproline was quantified colorimetrically (Sigma MAK008). All animal studies were approved by the University of Alabama at Birmingham Institutional Animal Care and Use Committee (protocol no. 23172).

### Human samples

Primary dermal fibroblasts were derived from patients with SSc at the University of Michigan (IRB HUM00065044) as previously described [2]. Lines included lcSSc, dcSSc, and healthy controls. Cells were used between passages 5–10.

### DNA and RNA isolation

Genomic DNA was extracted with the Monarch Genomic DNA Purification Kit (NEB 30102) with RNase A treatment. Total RNA was isolated with the Direct-zol RNA MicroPrep Kit (Zymo Research) with on-column DNase digestion. Concentrations were measured with a Qubit 4 Fluorometer (Thermo Fisher). DNA and RNA were stored at −80 °C until use. For each condition, material from ≥3 independent wells was pooled to minimize sampling variance.

### Centromere qPCR

Mouse centromeric arrays (MaSat, MiSat, and Ymin) were quantified by qPCR using validated primer sets [54]. Human α-satellite arrays (D1–D22, X, Y) were quantified using primer sets described previously [55]. Reactions were run on a QuantStudio 3 (Applied Biosystems) with Radiant SYBR Green Master Mix (Alkali Scientific). Reactions were performed in technical triplicates with ≤0.2 Ct variance. Melt-curve analysis verified single products. Relative copy number was normalized to 18S, GAPDH, or TOP3A using ΔΔCt method. Data were expressed as log fold change relative to vehicle controls.

### Western blotting

The cells were lysed in RIPA buffer (ChemCruz) supplemented with protease and phosphatase inhibitors (Thermo Fisher). Lysates (20–30 μg protein) were resolved by SDS–PAGE (10%) and transferred to PVDF membranes (Bio-Rad). Membranes were blocked in 5% milk/TBST, incubated overnight at 4 °C with primary antibodies (Supplementary Table 1), and probed with HRP-conjugated secondary antibodies (2 h, room temperature). Signals were developed using Clarity ECL substrate (Bio-Rad) and imaged with a ChemiDoc MP system. Band intensity was quantified in Fiji using rolling-ball background subtraction and normalized to β-actin.

### Immunofluorescence microscopy

Cells were fixed in 4% paraformaldehyde (PFA), permeabilized with PBST (0.2% Triton X-100), and blocked in 2% BSA/PBST. Coverslips were incubated with primary antibodies (Supplementary Table 1) for 1 h at 37 °C, followed by Alexa Fluor–conjugated secondary antibodies (1:1000, 45 min, room temperature). Nuclei were counterstained with ProLong Gold Antifade Mountant with DAPI.

Images were captured on an Olympus BX73 microscope (100× oil, NA 1.4) using identical exposure settings per experiment. Z-stacks (0.3 μm) were deconvolved in CellSens Dimension. Quantification of fluorescence intensity and micronuclei was performed in Fiji using automated thresholding and particle-analysis macros (n ≥ 50 fields per condition). Pearson’s r colocalization coefficients were calculated using Coloc2.

### Quantitative RT PCR

mRNA expression of DNA repair genes (RAD51 and KU70) was measured using the Luna One-Step RT-qPCR Kit (NEB) using 50 ng RNA per reaction on a QuantStudio 3 instrument. GAPDH was used as a reference gene. Triplicate biological replicates were run, and no-RT and no-template controls were included to exclude contamination. The sequences of primers used were obtained from published sources [56,57].

### Statistical analysis

All qPCR experiments were performed in three independent biological replicates with technical duplicates. Immunofluorescence analyses were based on ≥50 randomly selected fields per condition. Western blotting was performed in at least three independent replicates. Statistical significance was determined by two-tailed unpaired Student’s t-test or one-way ANOVA with Tukey’s or Dunnett’s post hoc test, as appropriate (GraphPad Prism v9.3.1). A p < 0.05 was considered significant. Data are presented as mean ± SD unless stated otherwise. The number of biological replicates (n) is indicated in figure legends.

## Supplementary Material

Supplement 1

Supplement 2

Additional information

Supplementary Information is available for this paper.

## References

[1] Volkmann ER, Andréasson K, Smith V. Systemic sclerosis. Lancet. 2023;401:304–18. doi:10.1016/S0140-6736(22)01692-0. 36442487 PMC9892343

[2] Paul S, Kaplan MH, Khanna D, McCourt PM, Saha AK, Tsou PS, Centromere defects, chromosome instability, and cGAS–STING activation in systemic sclerosis. Nat Commun. 2022;13:7074. doi:10.1038/s41467-022-34775-8. 36400785 PMC9674829

[3] van Bon L, Cossu M, Radstake TR. An update on an immune system that goes awry in systemic sclerosis. Curr Opin Rheumatol. 2011;23:505–10. doi:10.1097/BOR.0b013e32834b0dac. 21885976

[4] Rosendahl AH, Schönborn K, Krieg T. Pathophysiology of systemic sclerosis (scleroderma). Kaohsiung J Med Sci. 2022;38:187–95. doi:10.1002/kjm2.12505. 35234358 PMC11896191

[5] Kuwana M, Okano Y, Kaburaki J, Inoko H. HLA class II genes associated with anticentromere antibody in Japanese patients with systemic sclerosis (scleroderma). Ann Rheum Dis. 1995;54:983–7. doi:10.1136/ard.54.12.983. 8546531 PMC1010064

[6] Akbarali Y, Matousek-Ronck J, Hunt L, Staudt L, Reichlin M, Guthridge JM, Fine specificity mapping of autoantigens targeted by anti-centromere autoantibodies. J Autoimmun. 2006;27:272–80. doi:10.1016/j.jaut.2006.10.001. 17210244 PMC1906738

[7] Brown M, O’Reilly S. The immunopathogenesis of fibrosis in systemic sclerosis. Clin Exp Immunol. 2019;195:310–21. doi:10.1111/cei.13238. 30430560 PMC6378383

[8] Wei J, Bhattacharyya S, Tourtellotte WG, Varga J. Fibrosis in systemic sclerosis: emerging concepts and implications for targeted therapy. Autoimmun Rev. 2011;10:267–75. doi:10.1016/j.autrev.2010.09.015. 20863909 PMC3998379

[9] Ko J, Noviani M, Chellamuthu VR, Albani S, Low AHL. The pathogenesis of systemic sclerosis: the origin of fibrosis and interlink with vasculopathy and autoimmunity. Int J Mol Sci. 2023;24:14287. doi:10.3390/ijms241814287. 37762589 PMC10532389

[10] Usategui A, del Rey MJ, Pablos JL. Fibroblast abnormalities in the pathogenesis of systemic sclerosis. Expert Rev Clin Immunol. 2011;7:491–8. doi:10.1586/eci.11.39. 21790292

[11] Plikus MV, Wang X, Sinha S, Forte E, Thompson SM, Herzog EL, Fibroblasts: origins, definitions, and functions in health and disease. Cell. 2021;184:3852–72. doi:10.1016/j.cell.2021.06.024. 34297930 PMC8566693

[12] Kirsch-Volders M, Bolognesi C, Ceppi M, Bruzzone M, Fenech M. Micronuclei, inflammation and auto-immune disease. Mutat Res Rev. 2020;786:108335. doi:10.1016/j.mrrev.2020.108335. 33339583

[13] Aldrup-Macdonald ME, Sullivan BA. The past, present, and future of human centromere genomics. Genes (Basel). 2014;5:33–50. doi:10.3390/genes5010033. 24683489 PMC3966626

[14] Jeffery D, Lochhead M, Almouzni G. CENP-A: a histone H3 variant with key roles in centromere architecture in healthy and diseased states. Results Probl Cell Differ. 2022;70:221–61. doi:10.1007/978-3-031-06573-6_7. 36348109

[15] Logsdon GA, Ebert P, Audano PA, Loftus M, Porubsky D, Ebler J, Complex genetic variation in nearly complete human genomes. Nature. 2025;644:430–41. doi:10.1038/s41586-025-09140-6. 40702183 PMC12350169

[16] Vona R, Giovannetti A, Gambardella L, Malorni W, Pietraforte D, Straface E. Oxidative stress in the pathogenesis of systemic scleroderma: an overview. J Cell Mol Med. 2018;22:3308–14. doi:10.1111/jcmm.13630. 29664231 PMC6010858

[17] Kreuz S, Fischle W. Oxidative stress signaling to chromatin in health and disease. Epigenomics. 2016;8:843–62. doi:10.2217/epi-2016-0002. 27319358 PMC5619053

[18] Nassar R, Thompson L, Fouquerel E. Molecular mechanisms protecting centromeres from self-sabotage and implications for cancer therapy. NAR Cancer. 2023;5:zcad019. doi:10.1093/narcan/zcad019. PMC10167631 37180029

[19] McNulty SM, Sullivan BA. Alpha satellite DNA biology: finding function in the recesses of the genome. Chromosome Res. 2018;26:115–38. doi:10.1007/s10577-018-9582-3. 29974361 PMC6121732

[20] Black EM, Giunta S. Repetitive fragile sites: centromere satellite DNA as a source of genome instability in human diseases. Genes (Basel). 2018;9:615. doi:10.3390/genes9120615. 30544645 PMC6315641

[21] Fachinetti D, Han JS, McMahon MA, Ly P, Abdullah A, Wong AJ, DNA sequence-specific binding of CENP-B enhances the fidelity of human centromere function. Dev Cell. 2015;33:314–27. doi:10.1016/j.devcel.2015.03.020. 25942623 PMC4421092

[22] Krupina K, Goginashvili A, Cleveland DW. Causes and consequences of micronuclei. Curr Opin Cell Biol. 2021;70:91–9. doi:10.1016/j.ceb.2021.01.004. 33610905 PMC8119331

[23] Fenech M, Knasmueller S, Bolognesi C, Holland N, Bonassi S, Kirsch-Volders M. Micronuclei as biomarkers of DNA damage, aneuploidy, inducers of chromosomal hypermutation and as sources of pro-inflammatory DNA in humans. Mutat Res Rev. 2020;786:108342. doi:10.1016/j.mrrev.2020.108342. 33339572

[24] Zhang CZ, Spektor A, Cornils H, Francis JM, Jackson EK, Liu S, Chromothripsis from DNA damage in micronuclei. Nature. 2015;522:179–84. doi:10.1038/nature14493. 26017310 PMC4742237

[25] Mackenzie KJ, Carroll P, Martin CA, Murina O, Fluteau A, Simpson DJ, cGAS surveillance of micronuclei links genome instability to innate immunity. Nature. 2017;548:461–5. doi:10.1038/nature23449. 28738408 PMC5870830

[26] Harding SM, Benci JL, Irianto J, Discher DE, Minn AJ, Greenberg RA. Mitotic progression following DNA damage enables pattern recognition within micronuclei. Nature. 2017;548:466–70. doi:10.1038/nature23470. 28759889 PMC5857357

[27] Gourh P, Safran SA, Alexander T, Boyden SE, Morgan ND, Shah AA, HLA and autoantibodies define scleroderma subtypes and risk in African and European Americans and suggest a role for molecular mimicry. Proc Natl Acad Sci U S A. 2020;117:552–62. doi:10.1073/pnas.1906593116. 31871193 PMC6955366

[28] Furukawa H, Oka S, Kawasaki A, Shimada K, Sugii S, Matsushita T, Human leukocyte antigen and systemic sclerosis in Japanese: the sign of the four independent protective alleles. PLoS One. 2016;11:e0154255. doi:10.1371/journal.pone.0154255. PMC4846066 27116456

[29] Dengjel J, Schoor O, Fischer R, Reich M, Kraus M, Müller M, Autophagy promotes MHC class II presentation of peptides from intracellular source proteins. Proc Natl Acad Sci U S A. 2005;102:7922–7. doi:10.1073/pnas.0501190102. 15894616 PMC1142372

[30] Münz C. Antigen processing for MHC class II presentation via autophagy. Front Immunol. 2012;3:9. doi:10.3389/fimmu.2012.00009. 22566895 PMC3342365

[31] Gülle S, Çelik A, Birlik M, Yılmaz O. Skin and lung fibrosis induced by bleomycin in mice: a systematic review. Reumatismo. 2024;76:22–32. doi:10.4081/reumatismo.2024.1642. 38523580

[32] Chen J, Stubbe JA. Bleomycin: towards better therapeutics. Nat Rev Cancer. 2005;5:102–12. doi:10.1038/nrc1547. 15685195

[33] Murray V, Martin RF. The sequence specificity of bleomycin-induced DNA damage in intact cells. J Biol Chem. 1985;260:10389–91. doi:10.1016/S0021-9258(19)85092-5. 2411722

[34] Hecht SM. Bleomycin: new perspectives on the mechanism of action. J Nat Prod. 2000;63:158–68. doi:10.1021/np990549f. 10650103

[35] Chen J, Ghorai MK, Kenney G, Stubbe J. Mechanistic studies on bleomycin-mediated DNA damage: multiple binding modes can result in double-stranded DNA cleavage. Nucleic Acids Res. 2008;36:3781–90. doi:10.1093/nar/gkn302. 18492718 PMC2441780

[36] Murray V, Chen JK, Chung LH. The interaction of the metallo-glycopeptide anti-tumour drug bleomycin with DNA. Int J Mol Sci. 2018;19:1372. doi:10.3390/ijms19051372. 29734689 PMC5983701

[37] Bi X, Mills T, Wu M. Animal models in systemic sclerosis: an update. Curr Opin Rheumatol. 2023;35:364–70. doi:10.1097/BOR.0000000000000967. 37605874 PMC10553484

[38] Yoshizaki A, Yanaba K, Iwata Y, Komura K, Ogawa A, Akiyama Y, Cell adhesion molecules regulate fibrotic process via Th1/Th2/Th17 balance in a bleomycin-induced scleroderma model. J Immunol. 2010;185:2502–15. doi:10.4049/jimmunol.0901778. 20624949 PMC3733122

[39] Pawelec KM, Varnum M, Harkema JR, Auerbach B, Larsen SD, Neubig RR. Prevention of bleomycin-induced lung fibrosis via inhibition of the MRTF/SRF transcription pathway. Pharmacol Res Perspect. 2022;10:e01028. doi:10.1002/prp2.1028. PMC9695093 36426895

[40] Waseem M, Imtiaz A, Alexander A, Graham L, Contreras-Galindo R. Crosstalk between oxidative stress, mitochondrial dysfunction, chromosome instability, and the activation of the cGAS–STING/IFN pathway in systemic sclerosis. Ageing Res Rev. 2025;110:102812. doi:10.1016/j.arr.2025.102812. 40562314

[41] Her J, Bunting SF. How cells ensure correct repair of DNA double-strand breaks. J Biol Chem. 2018;293:10502–11. doi:10.1074/jbc.TM118.000371. 29414795 PMC6036189

[42] Aymard F, Bugler B, Schmidt CK, Guillou E, Caron P, Briois S, Transcriptionally active chromatin recruits homologous recombination at DNA double-strand breaks. Nat Struct Mol Biol. 2014;21:366–74. doi:10.1038/nsmb.2796. 24658350 PMC4300393

[43] Tsouroula K, Furst A, Rogier M, Heyer V, Maglott-Roth A, Ferrand A, Temporal and spatial uncoupling of DSB repair pathways within mammalian heterochromatin. Mol Cell. 2016;63:293–305. doi:10.1016/j.molcel.2016.06.002. 27397684

[44] Crow YJ, Manel N. Aicardi-Goutières syndrome and the type I interferonopathies. Nat Rev Immunol. 2015;15(7):429–40. 10.1038/nri3850. 26052098

[45] Gall A, Treuting P, Elkon KB, Loo YM, Gale M Jr, Barber GN, Autoimmunity initiates in nonhematopoietic cells and progresses via lymphocytes in an interferon-dependent autoimmune disease. Immunity. 2012;36(1):120–31. doi: 10.1016/j.immuni.2011.11.018. 22284419 PMC3269499

[46] Saayman X, Graham E, Nathan WJ, Nussenzweig A, Esashi F. Centromeres as universal hotspots of DNA breakage, driving RAD51-mediated recombination during quiescence. Mol Cell. 2023;83:523–38.e7. doi:10.1016/j.molcel.2023.01.004. 36702125 PMC10009740

[47] Zeitlin SG, Baker NM, Chapados BR, Soutoglou E, Wang JY, Berns MW, Double-strand DNA breaks recruit the centromeric histone CENP-A. Proc Natl Acad Sci U S A. 2009;106:15762–7. doi:10.1073/pnas.0908233106. 19717431 PMC2747192

[48] Mitra S, Gómez-Raja J, Larriba G, Dubey DD, Sanyal K. Rad51–Rad52 mediated maintenance of centromeric chromatin in Candida albicans. PLoS Genet. 2014;10:e1004344. doi:10.1371/journal.pgen.1004344. PMC3998917 24762765

[49] De Rop V, Padeganeh A, Maddox PS. CENP-A: the key player behind centromere identity, propagation, and kinetochore assembly. Chromosoma. 2012;121:527–38. doi:10.1007/s00412-012-0386-5. 23095988 PMC3501172

[50] Denais CM, Gilbert RM, Isermann P, McGregor AL, te Lindert M, Weigelin B, Nuclear envelope rupture and repair during cancer cell migration. Science. 2016;352:353–8. doi:10.1126/science.aad7297. 27013428 PMC4833568

[51] Raab M, Gentili M, de Belly H, Thiam HR, Vargas P, Jimenez AJ, ESCRT III repairs nuclear envelope ruptures during cell migration to limit DNA damage and cell death. Science. 2016;352:359–62. doi:10.1126/science.aad7611. 27013426

[52] Kono Y, Adam SA, Sato Y, Reddy KL, Zheng Y, Medalia O, Nucleoplasmic lamin C rapidly accumulates at sites of nuclear envelope rupture with BAF and cGAS. J Cell Biol. 2022;221:e202201024. doi:10.1083/jcb.202201024. PMC9617480 36301259

[53] Guey B, Wischnewski M, Decout A, Makasheva K, Kaynak M, Sakar MS, BAF restricts cGAS on nuclear DNA to prevent innate immune activation. Science. 2020;369:823–8. doi:10.1126/science.aaw6421. 32792394

[54] Benedetti F, Silvestri G, Denaro F, Finesso G, Contreras-Galindo R, Munawwar A, Mycoplasma DnaK expression increases cancer development in vivo upon DNA damage. Proc Natl Acad Sci U S A. 2024;121:e2320859121. doi:10.1073/pnas.2320859121. PMC10927570 38412130

[55] Contreras-Galindo R, Fischer S, Saha AK, Lundy JD, Cervantes PW, Mourad M, Rapid molecular assays to study human centromere genomics. Genome Res. 2017;27:2040–9. doi:10.1101/gr.219709.116. 29141960 PMC5741061

[56] Wang B, Hou D, Liu Q, Wu T, Guo H, Zhang X, Artesunate sensitizes ovarian cancer cells to cisplatin by downregulating RAD51. Cancer Biol Ther. 2015;16:1548–56. doi:10.1080/15384047.2015.1071738. 26176175 PMC5391513

[57] Lim JW, Kim H, Kim KH. Expression of Ku70 and Ku80 mediated by NF-κB and cyclooxygenase-2 is related to proliferation of human gastric cancer cells. J Biol Chem. 2002;277:46093–101. doi:10.1074/jbc.M206603200. 12324457

