# Centromere Instability Drives Chromosome Damage and Autoantigen Exposure in Systemic Sclerosis

**DOI:** 10.1101/2025.08.22.671795

**Published:** 2025-12-04

**Authors:** Azait Imtiaz, Mohammad Waseem, Hudson O’Neill, Christine M. Wright, Bo-Ruei Chen, Wioletta Czaja, Rafael Contreras-Galindo

**Affiliations:** 1 Department of Genetics, University of Alabama at Birmingham, Birmingham, Alabama, 35233, USA; 2 Department of Pediatrics, Washington University in St. Louis, MO, 63001, USA.

## Abstract

**Background:**

Centromere breakage has been associated with anti-centromere antibodies in systemic sclerosis (SSc), yet the origins of centromere damage and its link to immune activation remain unclear. The bleomycin induced fibrosis model is widely used as an experimental model of SSc. Here, we investigated whether bleomycin selectively disrupts centromeres and whether such damage contributes to chromosome instability and immunogenic chromatin mislocalization.

**Methods:**

We evaluated centromere integrity and downstream genome damage-response phenotypes across complementary systems: the bleomycin mouse model of skin fibrosis, hTERT-immortalized human fibroblasts, and primary dermal fibroblasts from SSc patients. Centromeric α-satellite copy number, DNA damage and repair markers, micronuclei, nuclear envelope rupture, and colocalization of centromeric chromatin with antigen presentation machinery were assessed by qPCR, RT-qPCR, and immunofluorescence microscopy.

**Results:**

Bleomycin induced selective depletion of α-satellite repeats at centromeres in mouse skin. In human fibroblasts, bleomycin generated double-strand breaks by γH2AX foci that preferentially colocalized with CENP-A-marked active centromeres and produced persistent centromere loss. Damage signaling required ATM and relied mainly on RAD51-mediated homologous-recombination repair, yet centromere restoration remained incomplete. Bleomycin also increased micronuclei, cytoplasmic centromeric foci, and BANF1-marked nuclear-envelope rupture, consistent with mis-segregation-driven chromatin leakage. CENP-B colocalized with HLA-DRB1 in bleomycin-treated fibroblasts and limited cutaneous SSc (lccSSc) fibroblasts, revealing centromere-derived antigen-presentation signatures.

**Conclusions:**

This study identifies active centromeres as selectively vulnerable and incompletely repaired targets of bleomycin-induced DNA damage and demonstrates that centromere instability produces mislocalized chromatin capable of engaging antigen-presentation pathways. These findings provide mechanistic insight into SSc autoantibody specificity and link genome instability to immune activation in fibrotic autoimmunity.

## Introduction

Systemic sclerosis (SSc), or scleroderma, is a chronic autoimmune disease characterized by vascular injury, immune dysregulation, and progressive fibrosis of the skin and internal organs^1–4^ . A defining clinical feature of limited cutaneous SSc (lcSSc) is the presence of anti-centromere antibodies (ACAs) directed against centromeric proteins such as CENP-A and CENP-B^5,6^. However, the origin of these autoantigens remains poorly understood. Although immune and fibrotic pathways are increasingly defined^7–10^, the genomic lesions that drive autoimmunity remain unclear. Centromeres, complex repetitive regions that govern chromosome segregation, represent a particularly vulnerable genomic compartment whose instability could promote exposure of normally sequestered chromatin antigens to the immune system^2,11^.

Human centromeres are composed of ~171-bp α-satellite monomers arranged into higher-order repeat (HOR) arrays. The repair dynamics of centromeric DSBs in fibrotic or autoimmune contexts remain poorly understood^12–14^. While centromeres are essential for accurate chromosome segregation, their repetitive nature, secondary structural features, and chromatin context make them prone to replication stress and DNA damage^15–19^. Such fragility positions the centromere as both a guardian and potential trigger of genome instability. Damage in these regions can disrupt kinetochore assembly, drive chromosomal missegregation, and generate micronuclei that serve as compartments for further DNA fragmentation and aberrant repair^16,20–22^. Beyond their structural role, centromeric sequences can therefore become active participants in the cellular stress response and, under chronic injury, potential sources of neoantigens.

Missegregated chromosomes frequently give rise to micronuclei that rupture, releasing DNA fragments into the cytoplasm. Cytosolic DNA can activate innate immune sensors, inducing interferon responses^2,23–25^. In fibroblasts, this process coincides with exposure of nuclear proteins such as CENP-A/B and engagement of MHC class II molecules, particularly HLA-DRB1 alleles associated with ACA-positive SSc^2,26–28^. These observations suggest that recurrent centromere injury could provide the physical substrate for autoantigen generation in SSc.

Bleomycin (BLM), a chemotherapeutic antibiotic and widely used experimental fibrosis inducer, generates reactive oxygen species that produce both single- and double-strand DNA breaks (DSBs)^29,30^. Early studies suggested that BLM can cleave repetitive DNA, including α-satellite sequences^31–33^. In vivo, BLM administration recapitulates key features of SSc, including dermal fibrosis, fibroblast activation, and extracellular matrix accumulation^34–36^. These properties make BLM a powerful model to test whether centromeric DNA damage drives chromosome instability (CIN), immune activation, and autoantibody generation in SSc^2,37^. We therefore hypothesized that centromeres are selective targets of BLM-induced DNA damage and that their imperfect repair initiates a cascade that leads to CIN and mislocalization of centromeric chromatin that enables immune recognition.

DSBs can be resolved by non-homologous end joining (NHEJ) or homologous recombination (HR), yet the latter often introduces deletions due to misaligned templates^38–40^.

Here, we used BLM-induced DNA damage in fibroblasts and in vivo skin fibrosis models to investigate centromere fragility in the context of SSc. Through α-satellite qPCR, immunofluorescence mapping, ATM inhibition, and patient fibroblast analyses, we demonstrate that BLM induces DSBs at active centromeres. These lesions are repaired primarily by RAD51-mediated HR but remain incompletely repaired and structurally unstable, resulting in DNA loss, kinetochore disruption, chromosome missegregation, and micronucleus formation. Centromeric chromatin becomes mislocalized to the cytoplasm, coinciding with BANF1 redistribution and colocalization of CENP-A/B with HLA-DRB1. Together, our findings support a centromere-first pathway linking genome instability to antigen exposure and provide a mechanistic molecular basis for ACA generation in SSc.

## Materials and methods

### Reagents

Bleomycin sulfate (Cayman Chemical, Cat. No. 13877, Lot #0701763–49) was used in all experiments to ensure consistency across assays. All other reagents were of analytical grade and obtained from commercial suppliers unless otherwise noted. Phosphate-buffered saline (PBS; pH 7.4), paraformaldehyde (PFA), and Triton X-100 were from Thermo Fisher. Bovine serum albumin (BSA; Sigma) was used for blocking at 2% (w/v). ProLong Gold Antifade Mountant with DAPI (Thermo Fisher) was used for all imaging.

### Cell culture and treatments

hTERT-immortalized human fibroblasts (CHON-002, ATCC CRL-2847; BJ-5ta, ATCC CRL-4001) were cultured in Dulbecco’s Modified Eagle Medium (DMEM; Gibco) supplemented with 10% fetal bovine serum (FBS; Gibco) and 1% penicillin–streptomycin (Gibco). The cells were maintained at 37 °C in a humidified 5% CO incubator and routinely tested for mycoplasma contamination.

For acute BLM exposure, fibroblasts were seeded at 5 × 10 cells per well in 6-well plates and grown to 60–80% confluence. The cells were treated with 0, 3.5, or 7 μM BLM for 3 h. For recovery assays, cells were washed with PBS and incubated in drug-free medium for 24 h. For ATM inhibition, cells were pretreated with KU-55933 (35 μM, Selleck Cat. S1092) for 1 h prior to BLM exposure.

### Mouse BLM-induced skin fibrosis model

C57BL/6 mice (Jackson Laboratory; 20–25 g; 2 males, 3 females per group) were randomized to receive intradermal injections of BLM (1 U/mL, 100 μL) or vehicle (PBS) into shaved dorsal skin every other day for 14 days. On day 14, mice were anesthetized with isoflurane (1–2%) and euthanized by cervical dislocation. Skin samples were collected from (1) PBS-injected control sites, (2) adjacent uninjected skin, and (3) fibrotic BLM-injected lesions. Dermal thickness was measured from H&E sections at 10 random fields per mouse using Fiji (ImageJ v1.54). Hydroxyproline was quantified colorimetrically (Sigma MAK008). All animal studies were approved by the University of Alabama at Birmingham Institutional Animal Care and Use Committee (protocol no. 23172).

### Human samples

Primary dermal fibroblasts were derived from patients with SSc at the University of Michigan (IRB HUM00065044) as previously described^2^. Lines included lcSSc, dcSSc, and healthy controls. Cells were used between passages 5–10.

### DNA and RNA isolation

Genomic DNA was extracted with the Monarch Genomic DNA Purification Kit (NEB 30102) with RNase A treatment. Total RNA was isolated with the Direct-zol RNA MicroPrep Kit (Zymo Research) with on-column DNase digestion. Concentrations were measured with a Qubit 4 Fluorometer (Thermo Fisher). DNA and RNA were stored at −80 °C until use. For each condition, material from ≥3 independent wells was pooled to minimize well-to-well variance.

### Centromere qPCR

Mouse centromeric arrays (MaSat, MiSat, and Ymin) were quantified by qPCR using validated primer sets^41^. Human α-satellite arrays (D1–D22, X, Y) were quantified using primer sets described previously^42^. Reactions were run on a QuantStudio 3 (Applied Biosystems) with Radiant SYBR Green Master Mix (Alkali Scientific). Reactions were performed in technical triplicates with ≤0.2 Ct variance. Melt-curve analysis verified single products. Relative copy number was normalized to 18S, GAPDH, or TOP3A using ΔΔCt method. Data were expressed as log fold change relative to vehicle controls.

### Western blotting

The cells were lysed in RIPA buffer (ChemCruz) supplemented with protease and phosphatase inhibitors (Thermo Fisher). Lysates (20–30 μg protein) were resolved by SDS–PAGE (10%) and transferred to PVDF membranes (Bio-Rad). Membranes were blocked in 5% milk/TBST, incubated overnight at 4°C with primary antibodies (Supplementary Table 1), and probed with HRP-conjugated secondary antibodies (2 h, room temperature). Signals were developed using Clarity ECL substrate (Bio-Rad) and imaged with a ChemiDoc MP system. Band intensity was quantified in Fiji using rolling-ball background subtraction and normalized to β-actin.

### Immunofluorescence microscopy

Cells were fixed in 4% paraformaldehyde (PFA), permeabilized with PBST (0.2% Triton X-100), and blocked in 2% BSA/PBST. Coverslips were incubated with primary antibodies (Supplementary Table 1) for 1 h at 37°C, followed by Alexa Fluor–conjugated secondary antibodies (1:1000, 45 min, room temperature). Nuclei were counterstained with ProLong Gold Antifade Mountant with DAPI.

Images were captured on an Olympus BX73 microscope (100× oil, NA 1.4) using identical exposure settings within each experiment. Z-stacks (0.3 μm) were deconvolved in CellSens Dimension. Quantification of fluorescence intensity and micronuclei was performed in Fiji using automated thresholding and particle-analysis macros (n ≥ 50 fields per condition). Pearson’s r colocalization coefficients were calculated using Coloc2.

### Quantitative RT PCR

mRNA expression of DNA repair genes (RAD51 and KU70) was measured using the Luna One-Step RT-qPCR Kit (NEB) using 50 ng RNA per reaction on a QuantStudio 3 instrument. GAPDH was used as a reference gene. Triplicate biological replicates were run, and no-RT and no-template controls were included as negative controls. The sequences of primers used were obtained from published sources^43,44^.

### Statistical analysis

All qPCR experiments were performed in three independent biological replicates with technical duplicates. Immunofluorescence analyses were based on ≥50 randomly selected fields per condition. Western blotting was performed in at least three independent replicates. Statistical significance was determined by two-tailed unpaired Student’s t-test or one-way ANOVA with Tukey’s or Dunnett’s post hoc test, as appropriate (GraphPad Prism v9.3.1). A p < 0.05 was considered significant. Data are presented as mean ± SD unless stated otherwise. The number of biological replicates (n) is indicated in figure legends.

## Results

### Centromere instability in BLM-induced SSc fibrosis models

**Figure 1.**
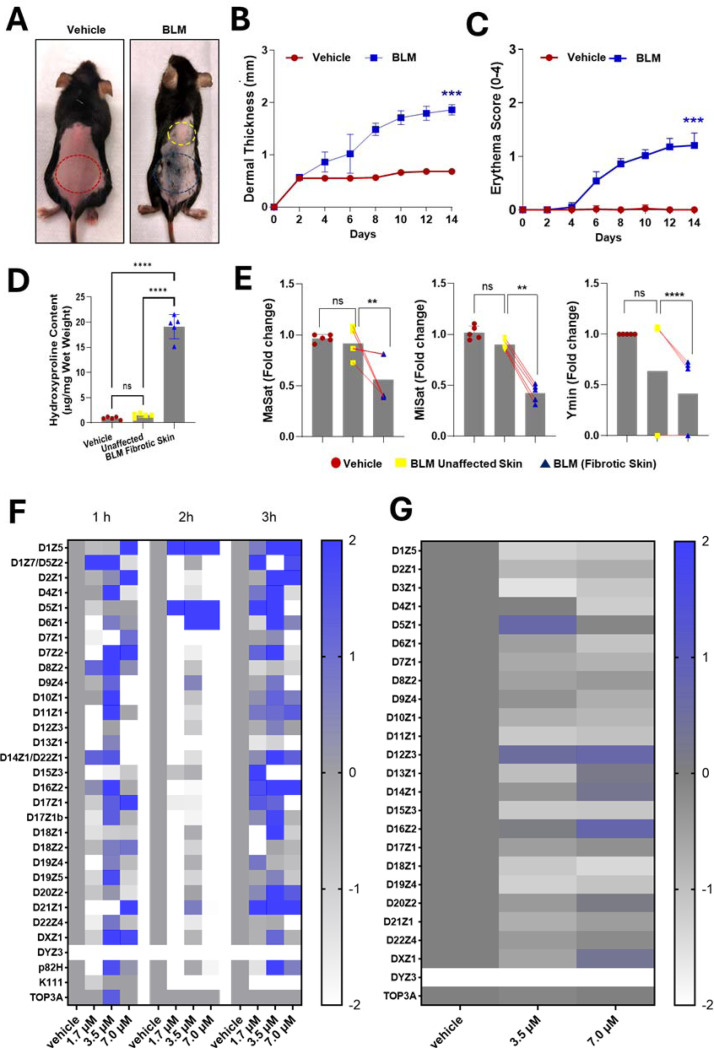
Heatmap and qPCR analysis of α-satellite array abundance in fibroblasts and mouse skin after bleomycin. (A) Representative dorsal skin images after 14 days of vehicle or BLM injections. Red indicates vehicle-treated skin, yellow marks adjacent skin, and blue indicates fibrotic lesions. (B, C) Dermal thickness and erythema scores after 14 days (n = 5 per group). (D) Hydroxyproline levels showing increased collagen in fibrotic skin compared with vehicle and adjacent regions. (E) qPCR of mouse α-satellite families (MaSat, MiSat, Ymin) normalized to 18S rRNA, demonstrating reduced repeat abundance in fibrotic skin; paired lines connect samples from the same mouse. (F) Heatmap of log fold changes in human α-satellite monomers in CHON-002 fibroblasts after 1–3 h of BLM. (G) Heatmap of α-satellite abundance after 3 h BLM followed by 24 h recovery. Color scale reflects Z-scores of log fold change (blue, gain; white, loss; gray, no change). DYZ3 is absent in CHON-002 cells. These data demonstrate that BLM induces fibrosis in mice and that human fibroblasts exhibit reduced α-satellite abundance after genotoxic stress, consistent with incomplete repair and centromere instability.. One-way ANOVA: **p < 0.01; ***p < 0.001; ****p < 0.0001; ns, not significant.

To test whether BLM induces centromere instability in a fibrosis-relevant context, we first used the intradermal BLM mouse skin model, which reproduces dermal thickening, collagen deposition, and fibroblast activation. The mice received BLM injections every other day for 14 days. By day 14, fibrotic skin showed marked dermal thickness, erythema, and hydroxyproline accumulation compared with vehicle control-treated skin (Figure 1A–D). Quantitative PCR (qPCR) of centromeric repeats revealed significant copy-number loss, with deletions of ~50% for MaSat, ~60% for MiSat, and ~70% for Ymin in fibrotic tissue (Figure 1E). These reductions were consistently observed in all biological replicates (n = 5 mice per group) and correlated with dermal thickness (r = 0.82, *p* < 0.01), indicating that centromere depletion correlates with fibrosis extent.

In human fibroblasts (CHON-002), BLM exposure (1.7–7.0 μM, 1–3 h) caused a rapid reduction in α-satellite DNA at 2 h, followed by partial recovery at 3 h, consistent with active repair processes (Figure 1F). At higher doses (7.0 μM), instability persisted. After 24 h of recovery, qPCR revealed sustained copy-number alterations (predominantly deletions, with occasional insertions) across multiple α-satellite arrays, including ~50% deletions at D1Z5, D6Z1, D15Z3, and D18Z1 (Figure 1G). BJ-5ta fibroblasts displayed a comparable pattern but with a greater magnitude of change (−0.8 to −1.5 log fold at several loci; Supplementary Figure 1A–B).

MTT assays confirmed ~75–80% viability, indicating that the observed changes reflect genomic instability rather than nonspecific cytotoxicity (Supplementary Figure 2). Together, these results establish that both *in vivo* and *in vitro* BLM exposure selectively destabilizes centromeric arrays while maintaining overall cell viability, providing a tractable model to dissect repair dynamics.

### BLM-induced DSBs at active centromeres

**Figure 2.**
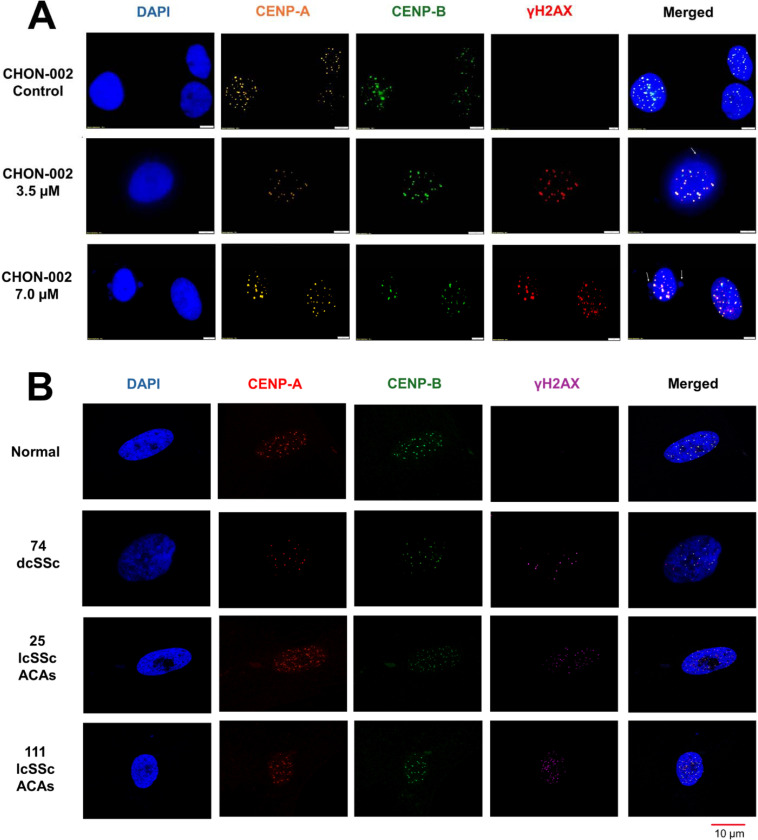
BLM-induced DNA damage at centromere loci in fibroblasts and SSc patient cells. (A) CHON-002 fibroblasts were treated with 3.5 or 7.0 μM BLM for 3 h and stained for CENP-A (orange), CENP-B (green), γH2AX (red), and DAPI (blue). Untreated cells displayed discrete nuclear CENP-A/B foci, whereas BLM exposure produced strong γH2AX staining colocalizing with centromeric regions (CENP-A), indicating accumulation of DSBs at active centromeres. (B) Primary dermal fibroblasts from healthy controls and from patients with diffuse cutaneous (dcSSc) or limited cutaneous (lcSSc) who were ACA-positive were stained for CENP-A (green) and γH2AX (purple) with DAPI (blue). Healthy fibroblasts exhibited few γH2AX foci, whereas lcSSc fibroblasts showed moderate γH2AX enrichment overlapping with centromeric domains, consistent with persistent centromeric DNA damage in disease cells. Together, these findings indicate that active centromeres are preferential targets of both BLM-induced and disease-associated DNA damage.

We next asked whether BLM preferentially induces DSBs at centromeres marked by CENP-A. Compared with untreated controls, CHON-002 fibroblasts treated with 3.5 or 7.0 μM BLM for 3 h showed strong γ-H2AX staining, whereas untreated controls showed no detectable signal (Figure 2A). A similar pattern was observed in BJ-5ta fibroblasts (Supplementary Figure 3A), and western blot analysis confirmed a robust, dose-dependent increase in γH2AX levels following BLM exposure (Supplementary Figure 3B).

Colocalization analysis revealed that γH2AX foci frequently overlapped with centromeric markers. While CENP-A and CENP-B signals remained tightly correlated (r > 0.85) under all conditions, γH2AX colocalized more strongly with CENP-A (r = 0.51–0.74) than with CENP-B (r = 0.31–0.50), indicating preferential damage at active centromeres (Supplementary Figure 3C–D). Double-positive γH2AX/CENP-A foci were not detected in untreated cells but became clearly visible after BLM treatment (*p* < 0.001, n = 50 cells), confirming centromere-locus specificity.

To assess disease relevance, we examined fibroblasts from three previously characterized SSc patients^2^. γH2AX activation was observed in all samples, consistent with earlier detection of γH2AX in nuclei and micronuclei in SSc fibroblasts^2^. In fibroblasts from two lcSSc patients, γH2AX colocalized with CENP-A in approximately half of the cells (Pearson’s r = 0.29–0.42), whereas colocalization with CENP-B was lower (r = 0.09–0.19). In contrast, fibroblasts from one dcSSc patient displayed γH2AX foci that rarely overlapped with CENP-A (<1% of cells; Figure 2B, Supplementary Figure 3E). These results extend prior observations by demonstrating that, in addition to γH2AX enrichment at micronuclei, DNA damage also accumulates at centromeric domains within nuclei of lcSSc fibroblasts. Together, these findings reveal subset-specific patterns of centromeric DNA damage, with ongoing active lesions in lcSSc fibroblasts and more complete structural loss in dcSSc.

### BLM-induced centromeric breaks are preferentially repaired by HR

**Figure 3.**
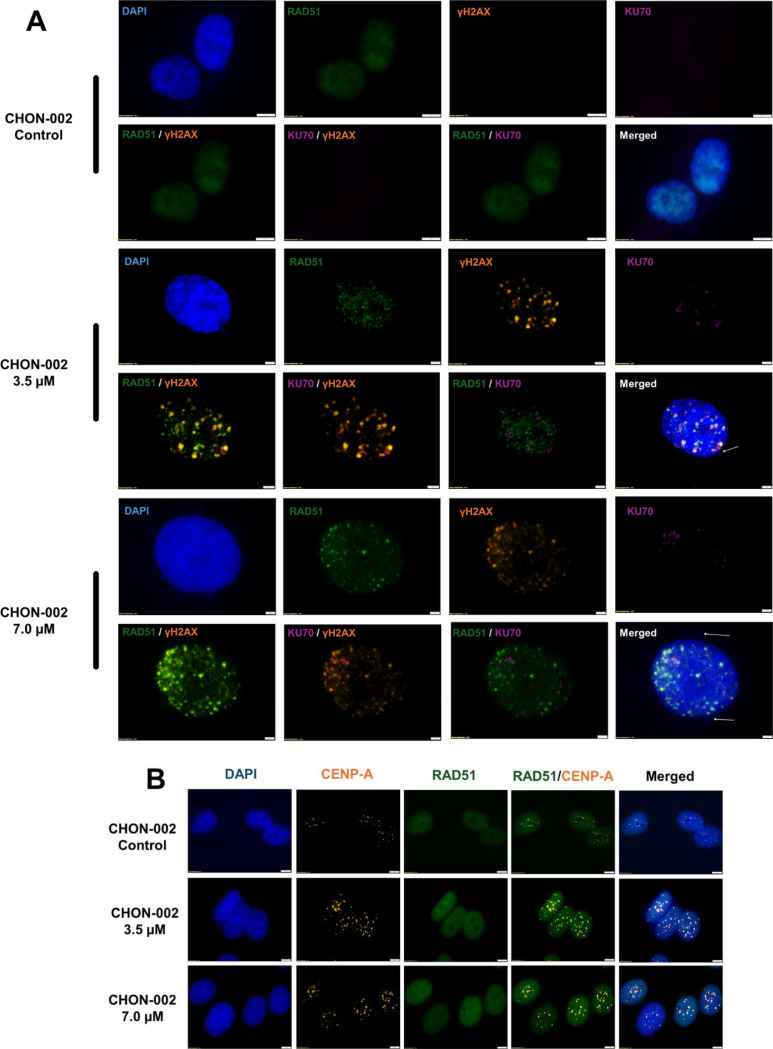
Centromeric DNA repair mechanisms after BLM treatment. CHON-002 fibroblasts were treated with 3.5 or 7.0 μM BLM for 3 h followed by 24 h recovery to assess activation of repair pathways at centromeric lesions. (A) Immunofluorescence (IF) staining for RAD51 (green; HR), γH2AX (orange; DSBs), and KU70 (purple; NHEJ). Untreated controls showed minimal nuclear staining, whereas BLM-treated cells exhibited strong γH2AX foci with prominent RAD51 recruitment at damage sites, indicating engagement of HR at centromeric breaks. White arrows denote micronuclei containing centromeric material. (B) Dual IF for CENP-A (orange) and RAD51 (green) after 3 h of BLM exposure revealed diffuse nuclear RAD51 prior to formation of discrete foci, consistent with early-stage HR activation prior to focus maturation. Together, these findings show that BLM-induced centromeric DSBs elicit robust HR activation, with limited KU70 engagement, and that incomplete repair may contribute to ongoing centromere instability.

To define the repair pathways that respond to centromere DSBs, CHON-002 fibroblasts were treated with 3.5 or 7.0 μM BLM for 3 h and stained for γH2AX (DSB marker), RAD51 (HR), and KU70 (NHEJ). Untreated cells showed minimal γH2AX signal, indicating the absence of spontaneous centromeric damage. After BLM treatment, strong nuclear γH2AX foci appeared throughout the nucleus, many overlapping with CENP-A–positive centromeres (Figure 3A). The γH2AX signal persisted following exposure, confirming the formation of centromeric DSBs that required active repair.

To determine how these lesions were resolved, we next examined RAD51 and KU70. RAD51 recruitment was readily detectable after BLM exposure and localized to the same nuclear regions marked by γH2AX, whereas KU70 staining remained weak and diffuse. Quantitative colocalization confirmed that HR was the predominant repair pathway. At 3.5 μM BLM, the correlation between RAD51 and γH2AX foci was high (r = 0.875), compared with a much lower correlation between KU70 and γH2AX (r = 0.314). At 7.0 μM, RAD51–γH2AX colocalization increased further (r = 0.919; Supplementary Figure 4A).

RT-qPCR showed strong RAD51 induction and modest KU70 changes (Supplementary Figure 4B). Over time, RAD51 exhibited a diffuse nuclear pattern at 3 h that resolved into discrete foci by 24 h (Figure 3B), consistent with ongoing HR and subsequent resolution of repair intermediates. Together, these findings demonstrate that BLM-induced DSBs at centromeres are recognized by the DNA damage response and repaired primarily through RAD51-dependent HR, with minimal engagement of the NHEJ pathway.

### BLM activates PARP-dependent single-strand break and replication stress signaling

To evaluate whether BLM also induces single-strand breaks (SSBs) and replication stress, we assessed accumulation of the poly(ADP-ribose) (PAR) polymer, a rapid and transient marker of PARP activation. In untreated CHON-002 fibroblasts, PAR immunofluorescence was minimal or undetectable, indicating low basal PARP activity (Supplementary Figure 4C). After exposure to 7.0 μM BLM, approximately 20% of cells displayed strong, pan-nuclear PAR staining, consistent with acute and transient PARP activation in response to DNA damage. The remaining population showed weak or absent PAR signal, reflecting the short half-life and rapid turnover of PAR polymers. Quantification confirmed a significant increase in nuclear PAR intensity compared with untreated controls (*p* < 0.0001; n = 50 cells; Supplementary Figure 4D).

Although PAR accumulation indicated activation of the SSB and replication stress response, the predominant DNA lesion induced by BLM was the double-strand break, as evidenced by the widespread γH2AX foci observed under the same conditions.

### ATM-mediated phosphorylation is required for γ-H2AX at centromeric DSBs

**Figure 4.**
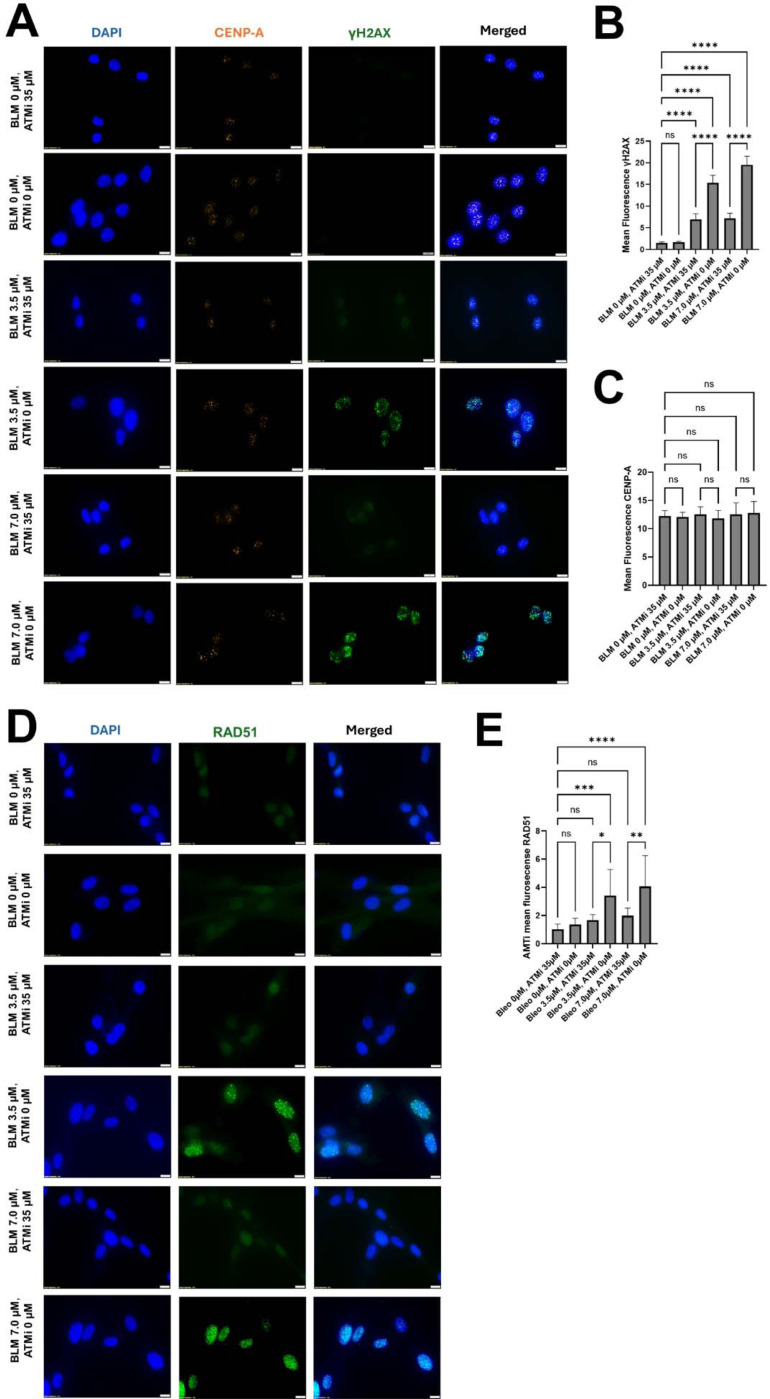
ATM inhibition reduces γH2AX and RAD51 accumulation at centromeres after BLM treatment. CHON-002 fibroblasts were treated with 3.5 or 7.0 μM BLM for 3 h with or without a 1 h pretreatment using the ATM inhibitor KU-55933 (35 μM). (A) Immunofluorescence (IF) for DAPI (blue), CENP-A (orange), and γH2AX (green; double-strand breaks). BLM exposure induced a dose-dependent increase in γH2AX foci that colocalized with CENP-A, indicating accumulation of DNA damage at active centromeres. ATM inhibition markedly reduced γH2AX accumulation without altering CENP-A localization or intensity. Scale bar, 10 μm. (B) Quantification mean nuclear γH2AX intensity demonstrating that the dose-dependent induction by BLM was significantly attenuated by ATM inhibition (n = 50 cells/condition). (C) Quantification of mean nuclear CENP-A intensity showing no significant change across treatments. (D) IF for DAPI (blue) and RAD51 (green; HR) demonstrated that ATM inhibition reduced RAD51 recruitment to DNA-damage sites. Scale bar, 10 μm. (E) Quantification mean nuclear RAD51 intensity confirmed that BLM-induced RAD51 accumulation was significantly suppressed by ATM inhibition (n = 50 cells/condition). Overall, ATM kinase activity is required for efficient γH2AX signaling and RAD51 recruitment at centromeric DSBs, linking ATM-dependent signaling to HR activation at repetitive DNA. Data represent mean ± SD. One-way ANOVA: **p < 0.01; ***p < 0.001; ****p < 0.0001; ns, not significant.

ATM phosphorylates histone H2AX on serine-139, generating γH2AX at sites of DSBs. To test whether ATM mediates the centromeric DNA damage signal induced by BLM, CHON-002 fibroblasts were treated with 3.5 or 7.0 μM BLM for 3 h, with or without pretreatment with the ATM inhibitor KU-55933 (35 μM, 1 h). BLM induced a robust, dose-dependent increase in nuclear γH2AX foci, many of which colocalized with the centromeric marker CENP-A (Figure 4A). Inhibition of ATM substantially reduced both the number and intensity of γH2AX foci at all doses, confirming that phosphorylation of H2AX at centromeric lesions depends on ATM activity. Importantly, KU-55933 treatment did not alter CENP-A localization or abundance (Figure 4C), indicating that loss of γH2AX reflected impaired signaling rather than centromere disassembly. Across three independent experiments, KU-55933 reduced nuclear γH2AX fluorescence by approximately 70% relative to BLM-treated controls (*p < 0.001, one-way ANOVA), establishing ATM as the principal kinase initiating the centromeric damage response.

### ATM signaling promotes RAD51 recruitment and HR repair

Because HR predominates at centromeric DSBs, we next examined whether ATM activity influences recruitment of the HR factor RAD51. After BLM exposure, numerous discrete RAD51 foci formed throughout the nucleus and frequently overlapped with γH2AX and CENP-A signals, consistent with assembly of HR repair complexes at damaged centromeres (Figure 4D). Pretreatment with KU-55933 sharply decreased both the number and fluorescence intensity of RAD51 foci and nearly eliminated their colocalization with γH2AX (Figure 4D–E). Quantification showed an average 65% reduction in nuclear RAD51 intensity and a three-fold decrease in cells exhibiting distinct RAD51 foci (*p* < 0.0001, n = 50 cells per condition). These data demonstrate that ATM not only generates the γH2AX signal but also facilitates efficient RAD51 recruitment to centromeric DSBs. In contrast, CENP-A intensity remained unchanged, underscoring that ATM activity modulates repair signaling rather than centromere identity.

Together, these findings define a two-step regulatory role for ATM at centromeric DSBs: first, phosphorylation of H2AX to initiate the DNA damage signal, and second, recruitment of RAD51 to ensure high-fidelity HR repair. Inhibition of ATM leaves centromeric lesions unrepaired, predisposing chromosomes to missegregation and micronucleus formation.

### BLM-induced DNA damage triggers micronuclei formation and centromere chromatin release

**Figure 5.**
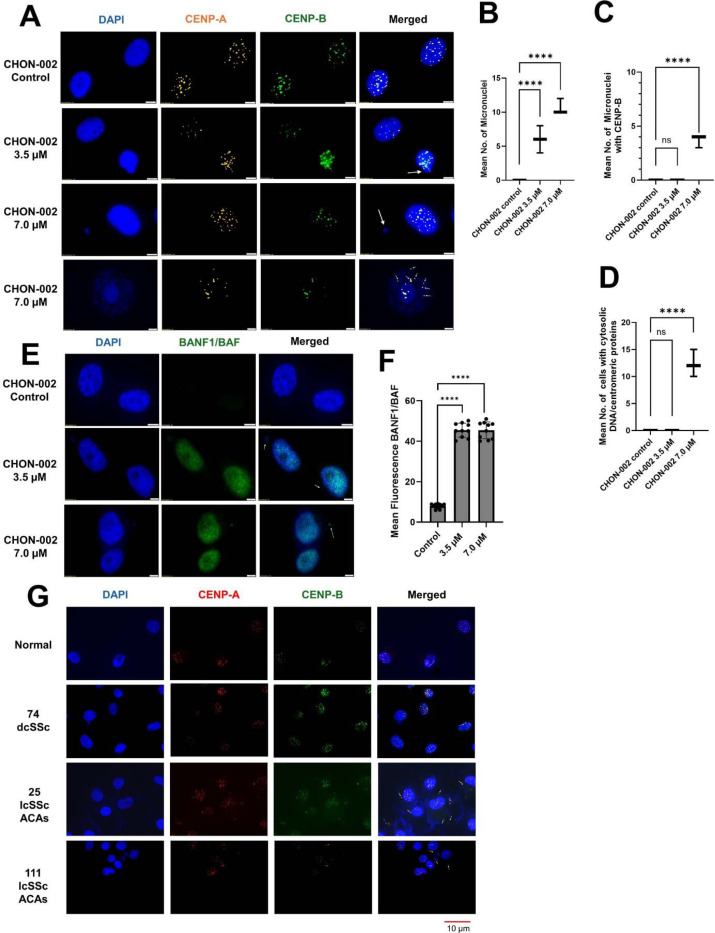
BLM induces micronuclei, cytoplasmic centromeric foci, and nuclear-envelope rupture in fibroblasts and SSc cells. (A) CHON-002 fibroblasts were treated with 3.5 or 7.0 μM BLM for 3 h followed by 24 h recovery and stained for CENP-A (orange), CENP-B (green), and DAPI (blue). Untreated control cells exhibited intact nuclei, whereas BLM exposure led to formation of micronuclei (white arrows) and cytoplasmic centromeric foci (arrowheads). Scale bar, 5 μm. (B–D) Quantification of (B) micronucleated cells, (C) micronuclei positive for CENP-A/B, and (D) cells containing cytoplasmic centromere-derived protein-DNA structures (mean ± SD three experiments, n = 100 cells/group). (E) Immunofluorescence for BANF1 (green) showed exclusive nuclear localization in control cells but redistribution to both nuclei and micronuclei after BLM treatment (white arrows), consistent with nuclear-envelope disruption. DAPI, blue. Scale bar, 5 μm. (F) Quantification of BANF1 fluorescence intensity (n = 10 cells/condition). (G) Primary fibroblasts from healthy controls and SSc patients stained for CENP-A (red), CENP-B (green), and DAPI (blue). Healthy fibroblasts displayed intact nuclei, whereas SSc fibroblasts exhibited micronuclei and cytoplasmic centromeric foci (white arrows). Scale bar, 10 μm. Overall, BLM-induced centromere instability promotes chromosome missegregation, micronucleus formation, and nuclear-envelope rupture, paralleling phenotypes observed in SSc fibroblasts. Data represent mean ± SD. One-way ANOVA: ****p < 0.0001; ns, not significant.

DAPI staining revealed a dose-dependent increase in micronuclei in CHON-002 fibroblasts treated with 3.5 or 7.0 μM BLM, whereas untreated controls showed no detectable micronuclei (Figure 5A–B). Many of these micronuclei contained centromeric signals identified by CENP-A and CENP-B immunostaining (Figure 5C, Supplementary Figure 5E), consistent with previous observations in SSc fibroblasts^2^. In untreated cells, CENP-A and CENP-B localized exclusively to nuclear centromeres, reproducing the organization seen in healthy skin fibroblasts^2^. After BLM exposure, discrete centromeric foci appeared in the cytoplasm, indicating mislocalization of centromeric chromatin. At 7.0 μM, approximately 12% of cells displayed cytoplasmic centromere chromatin (*p* < 0.0001, n = 3; Figure 5D), a finding also observed in BJ-5ta fibroblasts (Supplementary Figure 5A–D). Cytoplasmic DNA appeared in ~15% of cells, consistent with chromatin mislocalization.

To test whether nuclear envelope rupture mediates this release, we examined the localization of BANF1, which we previously showed to accumulate at rupture sites in lcSSc fibroblasts with cytoplasmic centromere leakage^2^. Consistent with those observations, BLM-treated fibroblasts displayed strong BANF1 accumulation on disrupted nuclei and micronuclei, supporting nuclear-envelope disruption as the source of centromere-containing chromatin mislocalization (Figure 5E–F).

Patient fibroblasts presented similar phenotypes^2^. Fibroblasts from lcSSc patients frequently displayed cytoplasmic centromeric chromatin, whereas dcSSc fibroblasts rarely did, matching ACA patterns^2^ (Figure 5G). Taken together, these data, along with BANF1 redistribution, implicate nuclear envelope rupture as a mechanism driving centromeric chromatin release in lcSSc fibroblasts.

### Centromere damage drives cytoplasmic mislocalization and MHC class II presentation

Cytoplasmic mislocalization of centromeric chromatin was a defining feature of fibroblasts treated with BLM, consistent with the phenotype previously documented in ACA-positive lcSSc fibroblasts^2^. In our earlier study, we detected cytoplasmic staining of the centromere proteins CENP-A and CENP-B in six of nine lcSSc patient fibroblast cultures, ranging from 4 to 78 percent of cells (n = 500). This phenotype correlated completely with the presence of ACAs and was absent in fibroblasts from healthy controls, dcSSc patients, and ACA-negative lcSSc fibroblasts. We now show that this mislocalization can be mechanistically reproduced by BLM-induced centromere damage in otherwise normal fibroblasts, indicating that genotoxic stress alone is sufficient to trigger cytoplasmic release of centromeric chromatin.

**Figure 6.**
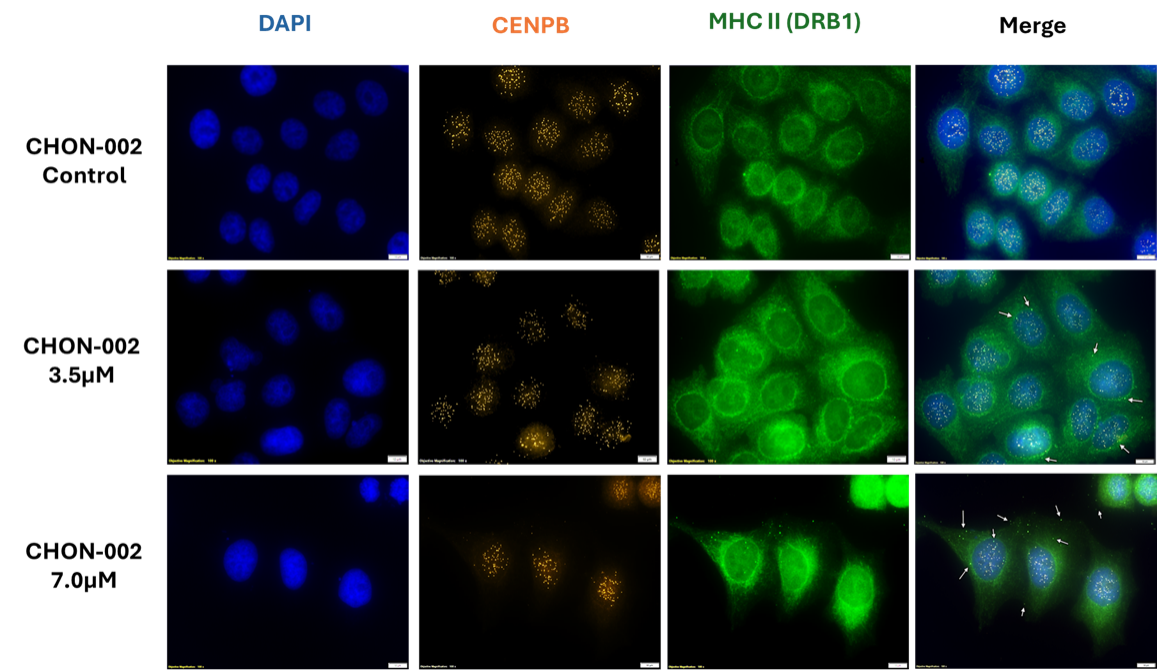
Colocalization of cytoplasmic CENP-B and MHC class II (DRB1) after BLM treatment. CHON-002 fibroblasts were left untreated or treated with 3.5 or 7.0 μM BLM for 3 h followed by 24 h recovery, then stained for CENP-B (orange) and MHC class II (DRB1, green) with DAPI (blue) marking nuclei. Untreated control cells showed no cytoplasmic overlap between CENP-B and MHC class II, whereas BLM-treated fibroblasts displayed distinct cytoplasmic regions where CENP-B and MHC class II colocalized (white arrows). Scale bar, 10 μm. These findings indicate that centromeric chromatin fragments released into the cytoplasm after DNA damage can associate with antigen-presentation machinery, providing a potential mechanistic link between centromere instability and immune activation.

To determine whether this cytoplasmic centromere material interacts with antigen presentation pathways, we performed immunofluorescence staining for CENP-B and MHC class II molecules (HLA-DRB1). BLM-treated fibroblasts exhibited strong cytoplasmic colocalization of CENP-B and HLA-DRB1, whereas untreated controls showed neither cytoplasmic centromere chromatin nor colocalization (Figure 6). The overlap was confined to cells displaying cytoplasmic CENP-B foci, consistent with a direct relationship between chromatin mislocalization and engagement of the antigen presentation machinery.

This phenomenon closely parallels what we observed in ACA-positive lcSSc fibroblasts, where CENP-B colocalized with HLA-DRB1 and HLA-DRB5 in the cytoplasm and along the plasma membrane. These interactions were absent in fibroblasts from dcSSc patients and healthy individuals^2^. The parallels between disease-derived cells and the BLM model indicate that persistent centromere damage and nuclear envelope rupture provide a physical route for centromere-derived chromatin to encounter MHC class II molecules.

Together, these findings show that BLM-induced centromere instability recapitulates a key immunopathological feature of lcSSc: the cytoplasmic exposure and potential MHC-II presentation of centromeric antigens. This establishes a direct mechanistic connection between genotoxic stress, nuclear rupture, and the initiation of centromere-targeted autoimmunity.

## Discussion

This study identifies centromeric DNA as a critical target of genotoxic stress in SSc. We demonstrate that BLM-induced breaks within centromeric α-satellite arrays are signaled through ATM-dependent γH2AX and repaired by RAD51-mediated HR, but lesions remain incompletely resolved, resulting in deletions or abnormal insertions. We observed DSBs across nearly all active CENP-A marked centromeres, indicating a genome-wide centromere response rather than isolated sequence-defined hotspots. This pattern suggests a chromatin-structure-dependent, non–sequence-specific cleavage mechanism reflecting the unique DNA topology of active centromeres. These findings clarify long-standing uncertainty about whether BLM targets α-satellite DNA in intact cells. A key consequence is cytoplasmic mislocalization of centromeric chromatin, where it colocalizes with MHC class II and may contribute to anti-centromere autoantigens. Parallel findings in SSc fibroblasts and the mouse fibrosis model reinforce centromere instability as a disease-relevant source of immune activation.

Our data support acute BLM exposure as a general model for studying centromere deletions and DNA-repair pathway choice. Because BLM induces DSBs across active centromeres without strong sequence bias, it enables investigation of how chromatin state, replication timing, and repair-factor availability influence deletion spectra and reintegration outcomes. Similar large-scale rearrangements arising from micronuclear fragmentation, including chromothripsis^23^, may share mechanistic overlap with centromere breakage. These properties make BLM a practical tool for dissecting how repetitive DNA regions undergo structural collapse under stress.

Our results reposition centromere instability as an active immunologic process rather than a passive structural defect. The observation that damaged centromere chromatin can exit the nucleus and associate with MHC class II machinery provides a direct link between chromosomal instability and systemic autoimmunity^45,46^. These events offer a mechanistic basis for ACA specificity and suggest that centromere-derived chromatin may directly engage antigenpresenting pathways in SSc.

Previous work shows that centromeres accumulate spontaneous lesions and are primarily repaired by RAD51-mediated HR^17,19,47^. In our experiments, BLM amplified this baseline fragility, producing deletions and insertions within α-satellite arrays. Alterations were more pronounced in BJ-5ta fibroblasts than in CHON-002 cells, suggesting differences in chromatin organization or repair capacity. The BLM-induced skin fibrosis model reproduced these effects *in vivo*, demonstrating reductions in mouse centromeric satellites within fibrotic tissue. These findings indicate that centromere instability is a defining molecular feature of fibrosis rather than a secondary, incidental consequence of DNA damage.

γH2AX foci colocalized with CENP-A, and ATM inhibition suppressed this signal, confirming ATM-dependent signaling. Recruitment of RAD51, but not KU70, indicated preference for HR, although this process did not fully restore array integrity^19,47,48^. Incomplete HR may underlie the α-satellite deletions detected in SSc fibroblasts, consistent with diminished centromere signal intensity in SSc skin biopsies^2^. Downstream consequences included chromosome missegregation, micronucleus formation, BANF1-marked nuclear rupture, and cytoplasmic export of centromeric chromatin—events reproduced in lcSSc fibroblasts.

These cytoplasmic structures appeared in ~12–15% of treated fibroblasts and mirrored lcSSc phenotypes. We observed cytoplasmic colocalization of CENP-B with HLA-DRB1, consistent with MHC-II engagement. These findings suggest that centromeric material released during nuclear rupture may directly encounter MHC-II complexes, providing a physical route for antigen presentation^2,5,50^.

Our conclusions rest on convergent evidence across qPCR, immunofluorescence, repair assays, and mouse skin analysis. Although acute BLM exposure does not model chronic remodeling, it provides a reproducible framework for dissecting centromere-specific injury. Future work should map breakpoints, define repair-driven deletion patterns, and determine how centromeric chromatin engages MHC-II and innate sensors^24^.

Our data support a centromere-first model in which repetitive DNA damage initiates fibrosis and autoimmunity. By demonstrating that BLM provokes widespread centromere-specific DSBs, followed by cytoplasmic export and MHC-II association, we provide an experimental system to study centromere deletions and their immunologic consequences. Understanding how conserved chromosomal regions become sources of immune activation may reveal therapeutic opportunities that enhance DNA-repair fidelity and protect chromatin integrity.

In summary, our findings show that centromeres are not passive structural elements but active sites of injury that can shape immune recognition in systemic sclerosis. The link between centromere breaks, micronuclei, nuclear envelope rupture, and cytoplasmic antigen exposure provides a mechanistic explanation for the specificity of anti-centromere antibodies and the distinct features of limited cutaneous disease. These results position centromere instability as a central driver of immune activation and fibrosis in SSc and highlight a pathway that may be relevant for patient stratification and future therapeutic targeting. Collectively, these insights refine our understanding of ACA-positive disease and identify centromere integrity as a potential therapeutic axis.

## Supplementary Material

Supplement 1

## References

[1] Volkmann ER, Andréasson K, Smith V. Systemic sclerosis. Lancet. 2023;401:304–18. doi:10.1016/S0140-6736(22)01692-0. 36442487 PMC9892343

[2] Paul S, Kaplan MH, Khanna D, McCourt PM, Saha AK, Tsou PS, Centromere defects, chromosome instability, and cGAS–STING activation in systemic sclerosis. Nat Commun. 2022;13:7074. doi:10.1038/s41467-022-34775-8. 36400785 PMC9674829

[3] van Bon L, Cossu M, Radstake TR. An update on an immune system that goes awry in systemic sclerosis. Curr Opin Rheumatol. 2011;23:505–10. doi:10.1097/BOR.0b013e32834b0dac. 21885976

[4] Rosendahl AH, Schönborn K, Krieg T. Pathophysiology of systemic sclerosis (scleroderma). Kaohsiung J Med Sci. 2022;38:187–95. doi:10.1002/kjm2.12505. 35234358 PMC11896191

[5] Kuwana M, Okano Y, Kaburaki J, Inoko H. HLA class II genes associated with anticentromere antibody in Japanese patients with systemic sclerosis (scleroderma). Ann Rheum Dis. 1995;54:983–7. doi:10.1136/ard.54.12.983. 8546531 PMC1010064

[6] Akbarali Y, Matousek-Ronck J, Hunt L, Staudt L, Reichlin M, Guthridge JM, Fine specificity mapping of autoantigens targeted by anti-centromere autoantibodies. J Autoimmun. 2006;27:272–80. doi:10.1016/j.jaut.2006.10.001. 17210244 PMC1906738

[7] Brown M, O’Reilly S. The immunopathogenesis of fibrosis in systemic sclerosis. Clin Exp Immunol. 2019;195:310–21. doi:10.1111/cei.13238. 30430560 PMC6378383

[8] Wei J, Bhattacharyya S, Tourtellotte WG, Varga J. Fibrosis in systemic sclerosis: emerging concepts and implications for targeted therapy. Autoimmun Rev. 2011;10:267–75. doi:10.1016/j.autrev.2010.09.015. 20863909 PMC3998379

[9] Ko J, Noviani M, Chellamuthu VR, Albani S, Low AHL. The pathogenesis of systemic sclerosis: the origin of fibrosis and interlink with vasculopathy and autoimmunity. Int J Mol Sci. 2023;24:14287. doi:10.3390/ijms241814287. 37762589 PMC10532389

[10] Usategui A, del Rey MJ, Pablos JL. Fibroblast abnormalities in the pathogenesis of systemic sclerosis. Expert Rev Clin Immunol. 2011;7:491–8. doi:10.1586/eci.11.39. 21790292

[11] Kirsch-Volders M, Bolognesi C, Ceppi M, Bruzzone M, Fenech M. Micronuclei, inflammation and auto-immune disease. Mutat Res Rev. 2020;786:108335. doi:10.1016/j.mrrev.2020.108335. 33339583

[12] Aldrup-Macdonald ME, Sullivan BA. The past, present, and future of human centromere genomics. Genes (Basel). 2014;5:33–50. doi:10.3390/genes5010033. 24683489 PMC3966626

[13] Jeffery D, Lochhead M, Almouzni G. CENP-A: a histone H3 variant with key roles in centromere architecture in healthy and diseased states. Results Probl Cell Differ. 2022;70:221–61. doi:10.1007/978-3-031-06573-6_7. 36348109

[14] Logsdon GA, Ebert P, Audano PA, Loftus M, Porubsky D, Ebler J, Complex genetic variation in nearly complete human genomes. Nature. 2025;644:430–41. doi:10.1038/s41586-025-09140-6. 40702183 PMC12350169

[15] Vona R, Giovannetti A, Gambardella L, Malorni W, Pietraforte D, Straface E. Oxidative stress in the pathogenesis of systemic scleroderma: an overview. J Cell Mol Med. 2018;22:3308–14. doi:10.1111/jcmm.13630. 29664231 PMC6010858

[16] Kreuz S, Fischle W. Oxidative stress signaling to chromatin in health and disease. Epigenomics. 2016;8:843–62. doi:10.2217/epi-2016-0002. 27319358 PMC5619053

[17] Nassar R, Thompson L, Fouquerel E. Molecular mechanisms protecting centromeres from self-sabotage and implications for cancer therapy. NAR Cancer. 2023;5:zcad019. doi:10.1093/narcan/zcad019. PMC10167631 37180029

[18] McNulty SM, Sullivan BA. Alpha satellite DNA biology: finding function in the recesses of the genome. Chromosome Res. 2018;26:115–38. doi:10.1007/s10577-018-9582-3. 29974361 PMC6121732

[19] Black EM, Giunta S. Repetitive fragile sites: centromere satellite DNA as a source of genome instability in human diseases. Genes (Basel). 2018;9:615. doi:10.3390/genes9120615. 30544645 PMC6315641

[20] Fachinetti D, Han JS, McMahon MA, Ly P, Abdullah A, Wong AJ, DNA sequence-specific binding of CENP-B enhances the fidelity of human centromere function. Dev Cell. 2015;33:314–27. doi:10.1016/j.devcel.2015.03.020. 25942623 PMC4421092

[21] Krupina K, Goginashvili A, Cleveland DW. Causes and consequences of micronuclei. Curr Opin Cell Biol. 2021;70:91–9. doi:10.1016/j.ceb.2021.01.004. 33610905 PMC8119331

[22] Fenech M, Knasmueller S, Bolognesi C, Holland N, Bonassi S, Kirsch-Volders M. Micronuclei as biomarkers of DNA damage, aneuploidy, inducers of chromosomal hypermutation and as sources of pro-inflammatory DNA in humans. Mutat Res Rev. 2020;786:108342. doi:10.1016/j.mrrev.2020.108342. 33339572

[23] Zhang CZ, Spektor A, Cornils H, Francis JM, Jackson EK, Liu S, Chromothripsis from DNA damage in micronuclei. Nature. 2015;522:179–84. doi:10.1038/nature14493. 26017310 PMC4742237

[24] Mackenzie KJ, Carroll P, Martin CA, Murina O, Fluteau A, Simpson DJ, cGAS surveillance of micronuclei links genome instability to innate immunity. Nature. 2017;548:461–5. doi:10.1038/nature23449. 28738408 PMC5870830

[25] Harding SM, Benci JL, Irianto J, Discher DE, Minn AJ, Greenberg RA. Mitotic progression following DNA damage enables pattern recognition within micronuclei. Nature. 2017;548:466–70. doi:10.1038/nature23470. 28759889 PMC5857357

[26] Gourh P, Safran SA, Alexander T, Boyden SE, Morgan ND, Shah AA, HLA and autoantibodies define scleroderma subtypes and risk in African and European Americans and suggest a role for molecular mimicry. Proc Natl Acad Sci U S A. 2020;117:552–62. doi:10.1073/pnas.1906593116. 31871193 PMC6955366

[27] Furukawa H, Oka S, Kawasaki A, Shimada K, Sugii S, Matsushita T, Human leukocyte antigen and systemic sclerosis in Japanese: the sign of the four independent protective alleles. PLoS One. 2016;11:e0154255. doi:10.1371/journal.pone.0154255. PMC4846066 27116456

[28] Dengjel J, Schoor O, Fischer R, Reich M, Kraus M, Müller M, Autophagy promotes MHC class II presentation of peptides from intracellular source proteins. Proc Natl Acad Sci U S A. 2005;102:7922–7. doi:10.1073/pnas.0501190102. 15894616 PMC1142372

[29] Gülle S, Çelik A, Birlik M, Yılmaz O. Skin and lung fibrosis induced by bleomycin in mice: a systematic review. Reumatismo. 2024;76:22–32. doi:10.4081/reumatismo.2024.1642. 38523580

[30] Chen J, Stubbe JA. Bleomycin: towards better therapeutics. Nat Rev Cancer. 2005;5:102–12. doi:10.1038/nrc1547. 15685195

[31] Murray V, Martin RF. The sequence specificity of bleomycin-induced DNA damage in intact cells. J Biol Chem. 1985;260:10389–91. doi:10.1016/S0021-9258(19)85092-5. 2411722

[32] Chen J, Ghorai MK, Kenney G, Stubbe J. Mechanistic studies on bleomycin-mediated DNA damage: multiple binding modes can result in double-stranded DNA cleavage. Nucleic Acids Res. 2008;36:3781–90. doi:10.1093/nar/gkn302. 18492718 PMC2441780

[33] Murray V, Chen JK, Chung LH. The interaction of the metallo-glycopeptide anti-tumour drug bleomycin with DNA. Int J Mol Sci. 2018;19:1372. doi:10.3390/ijms19051372. 29734689 PMC5983701

[34] Bi X, Mills T, Wu M. Animal models in systemic sclerosis: an update. Curr Opin Rheumatol. 2023;35:364–70. doi:10.1097/BOR.0000000000000967. 37605874 PMC10553484

[35] Yoshizaki A, Yanaba K, Iwata Y, Komura K, Ogawa A, Akiyama Y, Cell adhesion molecules regulate fibrotic process via Th1/Th2/Th17 balance in a bleomycin-induced scleroderma model. J Immunol. 2010;185:2502–15. doi:10.4049/jimmunol.0901778. 20624949 PMC3733122

[36] Pawelec KM, Varnum M, Harkema JR, Auerbach B, Larsen SD, Neubig RR. Prevention of bleomycin-induced lung fibrosis via inhibition of the MRTF/SRF transcription pathway. Pharmacol Res Perspect. 2022;10:e01028. doi:10.1002/prp2.1028. PMC9695093 36426895

[37] Waseem M, Imtiaz A, Alexander A, Graham L, Contreras-Galindo R. Crosstalk between oxidative stress, mitochondrial dysfunction, chromosome instability, and the activation of the cGAS–STING/IFN pathway in systemic sclerosis. Ageing Res Rev. 2025;110:102812. doi:10.1016/j.arr.2025.102812. 40562314

[38] Her J, Bunting SF. How cells ensure correct repair of DNA double-strand breaks. J Biol Chem. 2018;293:10502–11. doi:10.1074/jbc.TM118.000371. 29414795 PMC6036189

[39] Aymard F, Bugler B, Schmidt CK, Guillou E, Caron P, Briois S, Transcriptionally active chromatin recruits homologous recombination at DNA double-strand breaks. Nat Struct Mol Biol. 2014;21:366–74. doi:10.1038/nsmb.2796. 24658350 PMC4300393

[40] Tsouroula K, Furst A, Rogier M, Heyer V, Maglott-Roth A, Ferrand A, Temporal and spatial uncoupling of DSB repair pathways within mammalian heterochromatin. Mol Cell. 2016;63:293–305. doi:10.1016/j.molcel.2016.06.002. 27397684

[41] Benedetti F, Silvestri G, Denaro F, Finesso G, Contreras-Galindo R, Munawwar A, Mycoplasma DnaK expression increases cancer development in vivo upon DNA damage. Proc Natl Acad Sci U S A. 2024;121:e2320859121. doi:10.1073/pnas.2320859121. PMC10927570 38412130

[42] Contreras-Galindo R, Fischer S, Saha AK, Lundy JD, Cervantes PW, Mourad M, Rapid molecular assays to study human centromere genomics. Genome Res. 2017;27:2040–9. doi:10.1101/gr.219709.116. 29141960 PMC5741061

[43] Wang B, Hou D, Liu Q, Wu T, Guo H, Zhang X, Artesunate sensitizes ovarian cancer cells to cisplatin by downregulating RAD51. Cancer Biol Ther. 2015;16:1548–56. doi:10.1080/15384047.2015.1071738. 26176175 PMC5391513

[44] Lim JW, Kim H, Kim KH. Expression of Ku70 and Ku80 mediated by NF-κB and cyclooxygenase-2 is related to proliferation of human gastric cancer cells. J Biol Chem. 2002;277:46093–101. doi:10.1074/jbc.M206603200. 12324457

[45] Crow YJ, Manel N. Aicardi-Goutières syndrome and the type I interferonopathies. Nat Rev Immunol. 2015;15(7):429–40. 10.1038/nri3850. 26052098

[46] Gall A, Treuting P, Elkon KB, Loo YM, Gale M Jr, Barber GN, Autoimmunity initiates in nonhematopoietic cells and progresses via lymphocytes in an interferon-dependent autoimmune disease. Immunity. 2012;36(1):120–31. doi: 10.1016/j.immuni.2011.11.018. 22284419 PMC3269499

[47] Saayman X, Graham E, Nathan WJ, Nussenzweig A, Esashi F. Centromeres as universal hotspots of DNA breakage, driving RAD51-mediated recombination during quiescence. Mol Cell. 2023;83:523–38.e7. doi:10.1016/j.molcel.2023.01.004. 36702125 PMC10009740

[48] Zeitlin SG, Baker NM, Chapados BR, Soutoglou E, Wang JY, Berns MW, Double-strand DNA breaks recruit the centromeric histone CENP-A. Proc Natl Acad Sci U S A. 2009;106:15762–7. doi:10.1073/pnas.0908233106. 19717431 PMC2747192

[49] Mitra S, Gómez-Raja J, Larriba G, Dubey DD, Sanyal K. Rad51–Rad52 mediated maintenance of centromeric chromatin in Candida albicans. PLoS Genet. 2014;10:e1004344. doi:10.1371/journal.pgen.1004344. PMC3998917 24762765

[50] De Rop V, Padeganeh A, Maddox PS. CENP-A: the key player behind centromere identity, propagation, and kinetochore assembly. Chromosoma. 2012;121:527–38. doi:10.1007/s00412-012-0386-5. 23095988 PMC3501172

