# Supplementary material for: Centromere instability links genome damage to immune activation in systemic sclerosis
Source: bioRxiv. 2025 Oct 30:2025.08.22.671795. Preprint. [Version 4] doi: 10.1101/2025.08.22.671795 (PMC12636422; doi:10.1101/2025.08.22.671795)
Supplement: Supplementary file 2 [file media-2.docx]

| Target | Clone/Type | Host | Application/Dilution | Vendor/Cat. No. |
| --- | --- | --- | --- | --- |
| γ-H2AX (Ser139) | 20E3 | Rabbit | IF 1: 400; WB 1:1000 | CST #9718 |
| CENP-A | mAb | Mouse | 1:500 | MBL D115-3 |
| CENP-B | mAb | Mouse | 1:50 | SCBT sc-376392 |
| BANF1/BAF | EPR7668 | Rabbit | 1:100 | Abcam EPR7668 |
| MHCII DRB1 | mAb | Rabbit | 1:100 | Abclonal A7685 |
| RAD51 | mAb | Rabbit | 1:100 | Invitrogen PA5-27195 |
| KU70 | mAb | Rabbit | 1:100 | Invitrogen PA5-95270 |
| PAR | mAb | Mouse | 1:100 | SCBT sc-56198 |
| Actin B | AC-15 | Mouse | 1:5000 | Sigma-Aldrich A5441 |
| Alexa Fluor-conjugated secondary antibodies (488, 555, 568, 647; Invitrogen) were used at 1:1000. |  |  |  |  |

Supplementary Table 1. List of antibodies
